# Genome-wide host methylation profiling of anal and cervical carcinoma

**DOI:** 10.1371/journal.pone.0260857

**Published:** 2021-12-09

**Authors:** Erin M. Siegel, Abidemi Ajidahun, Anders Berglund, Whitney Guerrero, Steven Eschrich, Ryan M. Putney, Anthony Magliocco, Bridget Riggs, Kathryn Winter, Jeff P. Simko, Jaffer A. Ajani, Chandan Guha, Gordon S. Okawara, Ibrahim Abdalla, Mark J. Becker, Joseph F. Pizzolato, Christopher H. Crane, Kevin D. Brown, David Shibata

**Affiliations:** 1 Departments of Cancer Epidemiology, Tampa, FL, United States of America; 2 Department of Surgery, University of Tennessee Health Science Center, Memphis, TN, United States of America; 3 Biostatistics and Bioinformatics, Tampa, FL, United States of America; 4 Anatomic Pathology, H. Lee Moffitt Cancer Center & Research Institute, Tampa, FL, United States of America; 5 NRG Oncology Statistics and Data Management Center–ACR, Philadelphia, PA, United States of America; 6 UCSF Medical Center-Mount Zion, San Francisco, CA, United States of America; 7 M D Anderson Cancer Center, Houston, TX, United States of America; 8 Montefiore Medical Center, New York, NY, United States of America; 9 Juravinski Cancer Centre at Hamilton Health Sciences, Hamilton, ON, United States of America; 10 Cancer Research for the Ozarks CCOP, Springfield, MO, United States of America; 11 Columbus Community Clinical Oncology Program, Columbus, OH, United States of America; 12 Mount Sinai Comprehensive Cancer Center CCOP, Miami, FL, United States of America; 13 Department of Biochemistry and Molecular Biology, University of Florida, Gainesville, FL, United States of America; Istituto Nazionale Tumori IRCCS Fondazione Pascale, ITALY

## Abstract

HPV infection results in changes in host gene methylation which, in turn, are thought to contribute to the neoplastic progression of HPV-associated cancers. The objective of this study was to identify joint and disease-specific genome-wide methylation changes in anal and cervical cancer as well as changes in high-grade pre-neoplastic lesions. Formalin-fixed paraffin-embedded (FFPE) anal tissues (n = 143; 99% HPV+) and fresh frozen cervical tissues (n = 28; 100% HPV+) underwent microdissection, DNA extraction, HPV genotyping, bisulfite modification, DNA restoration (FFPE) and analysis by the Illumina HumanMethylation450 Array. Differentially methylated regions (DMR; t test q<0.01, 3 consecutive significant CpG probes and mean Δβ methylation value>0.3) were compared between normal and cancer specimens in partial least squares (PLS) models and then used to classify anal or cervical intraepithelial neoplasia-3 (AIN3/CIN3). In AC, an 84-gene PLS signature (355 significant probes) differentiated normal anal mucosa (NM; n = 9) from AC (n = 121) while a 36-gene PLS signature (173 significant probes) differentiated normal cervical epithelium (n = 10) from CC (n = 9). The CC progression signature was validated using three independent publicly available datasets (n = 424 cases). The AC and CC progression PLS signatures were interchangeable in segregating normal, AIN3/CIN3 and AC and CC and were found to include 17 common overlapping hypermethylated genes. Moreover, these signatures segregated AIN3/CIN3 lesions similarly into cancer-like and normal-like categories. Distinct methylation changes occur across the genome during the progression of AC and CC with overall similar profiles and add to the evidence suggesting that HPV-driven oncogenesis may result in similar non-random methylomic events. Our findings may lead to identification of potential epigenetic drivers of HPV-associated cancers and also, of potential markers to identify higher risk pre-cancerous lesions.

## Introduction

In the United States, approximately, 43,999 HPV-associated cancers are diagnosed annually and include malignancies of the oropharynx, anus, cervix, vulva, vagina, and penis [1, 2]. Cancers of the oropharynx, anus and vulva are among the handful of malignancies for which there continues to be a steady increase in incidence. For example, the incidence of anal cancer (AC) has jumped from 0.8 cases per 100,000 in 1975 to 1.8 cases per 100,000 per year based on 2010–2014 data [3]. Despite the availability of an HPV-targeted vaccine, these diseases continue to be a substantial burden to both US and world-wide populations due to low uptake and lag time of preventive effect [4].

The progression of normal epithelium through cervical intraepithelial neoplasia (CIN1 to CIN3), to cervical cancer (CC) is well described. Although approximately 30% of patients with untreated CIN3 will go on to develop invasive cancer, the vast majority do not [5]. AC is also known to develop through intraepithelial neoplasia (i.e., AIN1-3) and similar to CIN2/3 in cervical cancer, the majority of AIN3 lesions still do not progress to cancer. Given that these high grade anal and cervical lesions are routinely managed by surgical excision/ablation, a substantial number of patients are inherently overtreated. Consequently, the development of biomarkers for more selective treatment of high-risk premalignant lesions would be of significant clinical value [6].

DNA methylation is a key aberrant epigenetic event that has been documented in virtually every tumor type studied and is amongst the earliest disease-associated changes observed during tumorigenesis [7]. HPV may influence the host transcriptome through a number of epigenetic mechanisms including HPV E7 oncoprotein-mediated alterations in DNA methyltransferases [8, 9]. There is growing evidence to suggest that HPV-associated oncogenesis in different organ sites may be associated with common non-random genome-wide methylation events [10].

We and others have observed differential methylation patterns across the spectrum of anal squamous neoplastic progression including normal tissue, pre-cancerous lesions, and anal carcinoma [10–12]. Similarly, differential methylation of various genes is detected when comparing normal cervical tissue to CC, as well as to cytologically identified high-grade and low-grade intraepithelial lesions (HSIL, LSIL) [13, 14]. From a series of studies from the University of Amsterdam [15–18], using panels of 6 to 12 selected methylation markers derived from a methylation signature of cervical neoplasia, it has been suggested that both high-grade cervical and anal lesions may represent heterogeneous entities that harbor lower and higher risks for cancer progression [19]. In this study, exclusively using genome-wide methylation analyses, we sought to identify comprehensive methylomic profiles that differentiated anal or cervical cancer from normal mucosa, both disease specific and shared, and examined whether signatures derived from our methodologic approach were able to identify potential higher and lower risk high-grade anal and cervical lesions.

## Material and methods

### Anal and cervical tissues

Formalin-fixed paraffin-embedded (FFPE) anal tissues were identified from the NRG Oncology/ RTOG 98–11 biorepository at the UCSF Medical Center-Mount Zion. The 98–11 trial evaluated combinations of external beam irradiation plus chemotherapy in a phase III randomized controlled treatment trial of anal squamous cell carcinoma (SCC; concurrent 5-Fluorouracil (FU)/mitomycin-C vs. 5-FU/cisplatin) in patients enrolled between October 1998 and June 2005 [20]. Patients with a primary diagnosis of T1 or M1, severe comorbid conditions (including AIDS), or prior malignancy within the last 5 years were excluded [20].

For this study, archived FFPE tissue sections were obtained from patients in the Mitomycin-C arm of the trial. All sections were reviewed by two pathologists and regions of invasive AC, AIN3, and normal/benign anal mucosa were identified and microdissected. AIN3 and normal mucosa were identified in association with adjacent invasive ACs; however, AIN3 and normal tissues were frequently derived from separate paraffin blocks. In cases in which normal or AIN3 were on the same section, the tissues were clearly spatially delineated with confirmation by 2 independent pathologists and meticulously microdissected.

Under IRB-approved protocols, fresh frozen cervical tissues including normal cervix, CIN3, and invasive CCs were identified from the Moffitt Cancer Center Total Cancer Care Biorepository. Histology was confirmed by expert pathologists and tissues were microdissected. Ten benign/normal tissues, 9 CIN3 and 9 invasive CCs were obtained for this study. The 28 tissues were derived from 26 patients, of which 2 represented tumor-normal pairs. In some cases, CIN3 was identified adjacent to CCs.

### DNA processing

Genomic DNA from anal and cervical tissues was isolated using the QIAamp DNA extraction kit (QIAGEN, Valencia, CA). DNA concentrations were measured with picogreen-based Qubit® dsDNA HS Assay Kit (Invitrogen Cat # Q32851). Genomic DNA (500 ng) from cervical tissues underwent sodium bisulfite modification using the EZ DNA Methylation kit (Zymo Research, Orange, CA).

For anal tissues, DNA methylation quality of samples with sufficient amount (≥250ng) was assessed using the Illumina FFPE QC quantitative RT-PCR kit (Illumina, San Diego, CA) on the Applied Biosystems 7900HT platform. Eligible samples underwent sodium bisulfite modification using the EZ DNA Methylation kit followed by ligation using the Infinium HD FFPE DNA Restore kit (Illumina, San Diego, CA) as previously described [21].

### Methylation array

Genome-wide methylation was interrogated using the Infinium HumanMethylation450K BeadChip (HM450) following the manufacturer’s specifications which included whole-genome amplification, fragmentation, hybridization, base extension, counterstaining and scanning. A Tecan Liquid Handling robot with the Te-Flow apparatus was used for single base extension and staining, and chips were scanned on a single HiScanSQ System (Illumina Inc.). The HM450 incorporates both Infinium I (methylated and unmethylated beads per CpG locus) and Infinium II assays (one bead type with the methylated state determined at the single base extension step after hybridization) to evaluate the DNA methylation status at 485,512 CpG loci, which covers 99% of annotated genes and 96% of defined CpG islands [22–24].

### HPV genotyping

HPV status of anal and cervical DNA samples were determined using INNO-LiPA HPV Genotyping extra kit (Innogenetics, Gent, Belgium) which uses SPF10 consensus primers to amplify a 65bp biotinylated fragment from the HPV L1 region. Amplicons are then denatured and hybridized with specific oligonucleotide probes.

### Bioinformatics

Raw IDAT files were processed, background corrected and normalized using control probes in R using the minfi bioconductor package. β-values with a corresponding detection p-value>0.05 were set as missing and samples with >25% missing β-values were removed from the analysis. Methylation data from The Cancer Genome Atlas (TCGA) [25] for CCs were retrieved as raw IDATs and processed as described above. Gene expression data for CC and all other TCGA tumor types were downloaded from the Pan-Cancer Atlas at genomic data commons (GDC) (https://gdc.cancer.gov/about-data/publications/pancanatlas) and log2 transformed. All statistical tests were done using two-sided students t-test assuming unequal variance and false discovery corrected (q-value) as described by Storey [26]. A differentially methylated region (DMR) was defined as follows; Cervical: q-value<0.01, mean difference between groups > 0.4 and a minimum of four consecutive probes being significant within a gene. Anal: q-value<0.01, mean difference between groups > 0.3 and a minimum of three consecutive probes being significant within a gene. CpG definitions and gene models were taken from the Illumina manifest file. Partial Least Squares (PLS) using a binary response (1 = tumor, 0 = normal) was used for modeling the difference in DMR between normal and tumor samples [27]. Cross-validation was used to estimate the optimal number of PLS components [28]. The derived PLS models were also used to classify AIN3 in the anal tissue data set and CIN3 cases in the cervical tissue data set.

In addition, the CC progression PLS methylation model was applied for classification of normal, CIN3 and cervical tumors in four independent validation datasets: (a) HM450 BeadChip data from 307 tumor, 2 metastatic (Met) and 3 normal samples in TCGA CESC and (b) HM450 data obtained from 20 normal cervical tissues, 18 CIN3, and 6 CC samples and deposited within GEO GSE46306 [13] and (c) HM450 data from 28 normal, 36 CIN3 and for 4 tumor samples deposited in GSE99511 [18].

PCA models, PLS models, t-Distributed Stochastic Neighbor Embedding (t-SNE), methylation pattern across genes, and all statistical tests were done in MATLAB (Mathworks, Natick, MA).

## Results

### Tissues and demographics

From the 186 patients with available anal tissues, 121 invasive cases, 13 adjacent AIN3 and 9 adjacent normal mucosae yielded adequate amounts of genomic DNA (>250ng); thus, a total of 143 distinct samples were evaluated by methylation array. All cervical tissues including normal (n = 10), CIN3 (n = 9) and CC (n = 9) yielded adequate genomic DNA (>500ng) for methylation array analysis. All samples passed QC and β-value histograms are shown in **S1A and S1B Fig**.

The AC population consisted of 74 women and 47 men with a median age of 54 years (min-max:25–79). Cervical tissues were derived from 26 women with a median age of 35 years (min-max: 22–68). Race distribution was predominantly white for both the cervical (81%) and anal (87%) groups. Patient demographics are presented in **S1 Table**.

### HPV status

Of the 143 patient samples tested, 142 (99.3%) were positive for at least one or more HPV types while only one was negative for HPV. All 28 (100%) cervical specimens tested were HPV positive.

### PCA of methylomic alterations

**Fig 1:**
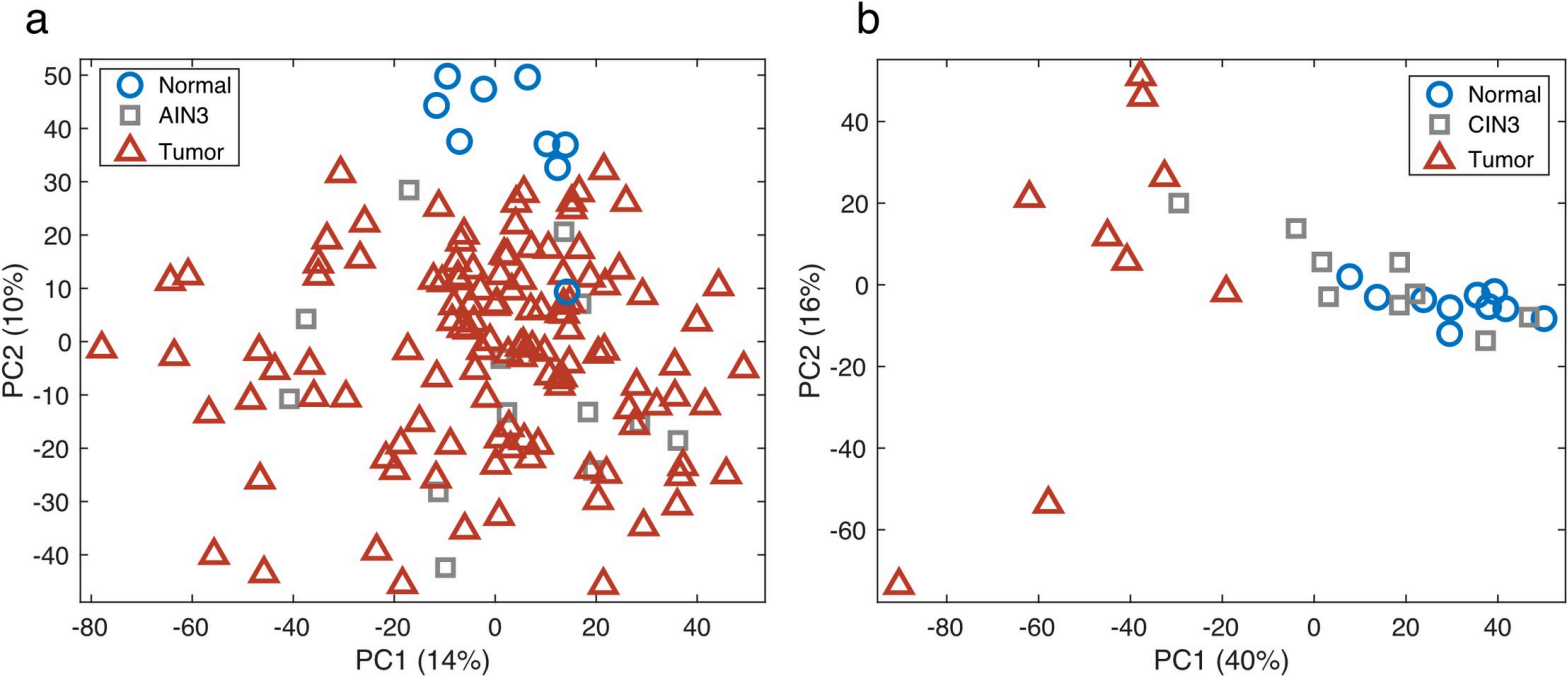
Principal Components Analysis (PCA) score plot. The PCA model, using >300, 000 beta-values, separates normal tissues (blue circles) from tumor samples (red triangles) for both AC (**a**) and CC (**b**). AIN3 samples (a, grey squares) clustered more with the tumor samples, whereas CIN3 samples (b, grey squares) segregated into those similar to normal epithelium and others more similar to cancer.

PCA was used to compare β-values for all 143 anal tissues (121 tumor, 13 AIN3 and 9 normal) across all probes (**Fig 1A)**. A separation between the normal anal mucosae (blue circles) and the ACs (red triangles) can be seen in the second PCA component. There were no strong outliers or batch effects observed. AIN3 cases (*in-situ* cancer; grey squares), which were all derived in association with AC, were all closely clustered with ACs. In addition to PCA, t-SNE was used to cluster the samples and separation of normal from AC was also observed (**S2A and S2B Fig**).

PCA was used to compare the methylation β-values for the 28 cervical tissues (9 tumor, 9 CIN3 and 10 normal) across all probes (**Fig 1B)**. A separation between the normal cervical tissue (blue circles) and CCs (red triangles) can be seen in the first PCA component. As with the anal tissue analyses, no strong outliers or batch-effects were observed. In contrast to the anal tissue analysis, CIN3s (grey squares), which were derived independently from CCs, segregated into those similar to normal epithelium and others more similar to cancer.

### Identification of DMRs in AC and CC

Using DMR-defining criteria, the comparison of normal anal mucosae with ACs resulted in 355 significant CpG loci representing 84 significant genes (**S2 Table**). For the cervical analysis, 36 genes, comprised of 173 CpG loci, were identified that significantly distinguished CC from normal cervical tissue (**S3 Table**). There were 17 overlapping genes total between the two panels. These genes and their biological functions are listed in **Table 1**. From this panel, we have arbitrarily selected 2 representative genes, ZIK1 and ASCL1 (previously identified as being differentially methylated in HPV-associated cancers), for expanded analyses [13].

**Table 1:** AC and CC overlapping methylated genes.

Gene Symbol	Product	Function/Association with Disease	CpG Number ^a^	Mean Beta value in Cervix				Mean Beta value in Anus				Known Epigenetic/Methylation association with Disease	Genomic Region	Directionality
				Normal	Cx SCC	Dif^b^	q-value^c^	Normal	Anal SCC	Dif^b^	q-value^c^			
ASCL1	Achaete-scute family bHLH transcription factor 1. ***Other aliases***: BHLHa46; ASH-1;	Transcription factor: accessing closed chromatin and activate neural pathways. Essential for neurons. Mutation in Neuroblastoma.	cg02246645	0.053	0.461	0.408	9.8E-04	0.138	0.482	0.344	1.4E-04	Among a panel of methylated genes for the detection of oral SCC	1stExon	Hypermethylated
			cg27420520	0.007	0.422	0.415	9.2E-04	0.074	0.408	0.333	4.6E-05			
			cg20718350	0.026	0.529	0.503	4.3E-04	0.158	0.509	0.351	5.3E-04			
			cg03700449	0.054	0.527	0.473	4.0E-04	0.144	0.507	0.363	3.6E-05			
			cg22356339	0.036	0.520	0.484	6.9E-06	0.143	0.420	0.276	6.4E-05			
ATP10A	ATPase phospholipid transporting 10A (putative)	It is a protein coding gene. Diseases associated with ATP10A include Angelman Syndrome and Angelman Syndrome Due to Imprinting Defect In 15Q11-Q13. Possible imprinted gene.	cg20174066	0.119	0.565	0.446	3.5E-04	0.276	0.509	0.232	8.8E-04	Modification of CpG sites in ATP10A genes after hypocaloric-diet-induced weight loss.	1stExon; 5’UTR; TSS200	Hypermethylated
			cg03419058	0.028	0.568	0.540	7.5E-04	0.212	0.587	0.375	3.7E-04			
			cg16389285	0.005	0.407	0.401	1.2E-03	0.101	0.401	0.299	9.4E-05			
			cg20124450	0.019	0.463	0.444	8.5E-04	0.159	0.467	0.307	4.7E-04			
			cg22113930	0.043	0.516	0.473	1.2E-03	0.215	0.522	0.307	3.0E-04			
			cg26230285	0.012	0.497	0.484	7.5E-04	0.163	0.521	0.358	3.4E-04			
CCDC81	Coiled-Coil Domain-Containing Protein 81	It is a Protein Coding gene associated with Brain Cancer; specifically, Ganglioneuroblastoma.	cg18282849	0.060	0.584	0.524	7.5E-06	0.176	0.498	0.323	1.7E-07	Hypermethylated in Endometrial Tumors	1stExon; 5’UTR; TSS200	Hypermethylated
			cg10395685	0.121	0.546	0.425	2.7E-06	0.266	0.577	0.310	2.9E-06			
			cg18121003	0.141	0.672	0.531	8.9E-07	0.264	0.564	0.301	3.8E-07			
			cg23817893	0.159	0.635	0.476	1.6E-07	0.311	0.596	0.285	1.5E-05			
			cg04573398	0.426	0.832	0.406	1.0E-05	0.754	0.830	0.076	9.2E-03			
DPP10	Dipeptidyl Peptidase Like 10. ***Other aliases*:** DP IV-related protein 3 (DPRP3)	May be associated with colorectal cancer. Seen in Autism, mood disorder or schizophrenia.	cg04075191	0.137	0.593	0.456	3.1E-04	0.225	0.558	0.333	3.9E-06	Methylated in nicotine-exposed fetal lung and placental tissue.	Body;1stExon;5’UTR	Hypermethylated
			cg01718116	0.124	0.547	0.424	5.2E-05	0.237	0.579	0.342	2.6E-05			
			cg00089091	0.188	0.617	0.429	1.3E-06	0.353	0.680	0.326	7.6E-05			
			cg13777681	0.039	0.582	0.543	5.2E-04	0.185	0.670	0.486	1.2E-04			
FMN2	Formin 2	Critical regulator of p21/cell cycle progression	cg15748490	0.054	0.432	0.379	2.8E-03	0.143	0.466	0.323	3.5E-06	Associated with parathyroid glioblastoma, retinoblastoma chondrosarcoma.	5’UTR; 1stExon; TSS200; TSS1500	Hypermethylated
			cg19591056	0.068	0.556	0.488	8.5E-05	0.231	0.564	0.333	5.9E-06			
			cg01535698	0.021	0.442	0.421	1.4E-04	0.147	0.486	0.339	4.1E-04			
			cg02574509	0.054	0.458	0.404	2.4E-04	0.135	0.420	0.285	2.8E-05			
			cg25208017	0.017	0.592	0.576	3.5E-05	0.147	0.607	0.460	1.2E-04			
MARCH11	Membrane associated ring-CH-type finger 11; E3 ubiquitin-protein ligase MARCH11	Modulates lysosomal degradation and delivery. Member of membrane-bound E3 ubiquitin ligases known to add ubiquitin to target lysines in substrate proteins, thereby signaling their intracellular transport.	cg25092681	0.044	0.444	0.400	2.3E-04	0.142	0.466	0.324	2.6E-05	Could not find associated disease information on MARCH11.	TSS200; TSS1500	Hypermethylated
			cg00339556	0.031	0.551	0.519	1.3E-05	0.165	0.460	0.295	8.6E-05			
			cg01791874	0.048	0.571	0.523	1.5E-05	0.189	0.548	0.360	2.7E-05			
			cg17030173	0.151	0.665	0.514	6.3E-07	0.293	0.588	0.295	1.1E-04			
			cg17712694	0.091	0.673	0.583	8.0E-08	0.264	0.605	0.341	3.3E-04			
			cg16150752	0.068	0.672	0.603	3.2E-07	0.264	0.622	0.358	6.3E-04			
			cg21901718	0.047	0.561	0.514	1.9E-05	0.182	0.496	0.314	1.9E-04			
			cg18325622	0.115	0.571	0.456	1.3E-07	0.267	0.584	0.317	1.2E-04			
			cg23065934	0.066	0.573	0.507	4.5E-06	0.268	0.596	0.328	8.7E-05			
MIR129-2	microRNA 1292	Short non-coding RNAs that are involved in post-transcriptional regulation of gene expression in multicellular organisms by affecting both the stability and translation of mRNAs.	cg15556502	0.013	0.488	0.475	9.6E-06	0.142	0.550	0.408	1.7E-05	Tumor suppressive methylated in lymphoid malignancies. associated with Univentricular Heart disorder.	TSS200; Body	Hypermethylated
			cg14416371	0.039	0.643	0.604	1.2E-06	0.260	0.693	0.433	1.4E-04			
			cg14944647	0.044	0.478	0.434	4.2E-06	0.151	0.525	0.374	2.4E-05			
			cg01939477	0.028	0.606	0.578	2.2E-06	0.181	0.618	0.437	1.1E-04			
			cg16407471	0.096	0.581	0.486	3.4E-06	0.243	0.615	0.371	6.9E-05			
			cg05376374	0.034	0.603	0.569	1.7E-07	0.162	0.546	0.384	1.6E-04			
			cg03365311	0.075	0.516	0.441	1.6E-05	0.195	0.520	0.325	1.6E-05			
PAX1	Paired box 1. ***Other aliases*:** Paired Domain Gene HuP48 (HuP48), OFC2	It is a member of the paired box (PAX) family of transcription factors and they play critical roles during vertebral column and fetal development.	cg19054524	0.054	0.504	0.449	2.7E-05	0.141	0.452	0.311	5.2E-05	Frequent methylation in HNSCC, silenced by methylation in ovarian and CCs	TSS200; 1stExon; 5’UTR	Hypermethylated
			cg08448701	0.065	0.514	0.450	2.4E-05	0.149	0.457	0.308	2.1E-05			
			cg01783070	0.059	0.570	0.511	1.4E-05	0.149	0.507	0.358	1.9E-05			
			cg19079845	0.103	0.537	0.434	5.0E-06	0.191	0.494	0.302	1.1E-05			
PEX5L	Peroxisomal biogenesis factor 5 like. ***Other aliases*:** TRIP8b, PEX5R, PXR2, PEX5-Related Protein,	Trafficking of peroxisomal matrix proteins	cg02009585	0.119	0.570	0.450	1.5E-05	0.264	0.579	0.315	1.1E-03	Rhizomelic Chondrodysplasia Punctata, Type 5 and Rhizomelic Chondrodysplasia Punctata, Type 2	TSS200; TSS1500	Hypermethylated
			cg23346462	0.075	0.594	0.519	8.4E-07	0.277	0.609	0.331	1.5E-03			
			cg02119363	0.054	0.496	0.443	1.0E-05	0.218	0.483	0.265	8.7E-04			
			cg13473356	0.085	0.542	0.457	2.4E-06	0.268	0.525	0.257	9.0E-04			
			cg04894619	0.104	0.551	0.447	7.2E-07	0.274	0.497	0.223	1.9E-03			
			cg18780412	0.030	0.559	0.529	6.5E-05	0.209	0.537	0.329	7.9E-04			
			cg21176048	0.023	0.424	0.401	3.6E-04	0.173	0.504	0.331	4.3E-04			
			cg05131623	0.151	0.629	0.477	2.9E-05	0.379	0.702	0.324	1.4E-03			
RYR2	Ryanodine receptor 2. ***Other aliases***: ARVD2, RYR-2, ARVC2, RyR2, VTSIP2.	Encodes a ryanodine receptor found in cardiac muscle sarcoplasmic reticulum. Mediates cellular calcium release	cg07790615	0.045	0.565	0.520	1.4E-04	0.218	0.617	0.398	6.2E-07	Associated with arrhythmogenic right ventricular dysplasia and ventricular tachycardia stress-induced polymorphic 1	TSS1500, Body	Hypermethylated
			cg03422911	0.258	0.673	0.415	7.7E-06	0.457	0.701	0.243	1.5E-04			
			cg18375860	0.081	0.513	0.432	1.4E-07	0.207	0.537	0.330	1.7E-04			
			cg11657808	0.117	0.586	0.469	7.8E-06	0.294	0.624	0.330	8.8E-05			
			cg07914084	0.057	0.547	0.490	9.0E-05	0.207	0.565	0.358	1.8E-04			
SORCS3	Sortilin related VPS10 domain containing receptor 3. ***Other aliases*:** KIAA1059, SORCS	Protein Coding, developing and maturation of the central nervous system. mediate transport from endoplasmic reticulum to Golgi.	cg08495770	0.105	0.544	0.439	2.5E-05	0.265	0.563	0.298	4.3E-05	Frequently methylated in gastric cancer. Associated with Alzheimer Disease.	TSS200, 1stExon; 5’UTR	Hypermethylated
			cg16787600	0.071	0.547	0.476	1.3E-05	0.213	0.533	0.320	7.8E-05			
			cg10778841	0.082	0.574	0.492	3.7E-07	0.209	0.541	0.332	1.4E-04			
			cg18326021	0.052	0.479	0.427	1.1E-05	0.166	0.471	0.305	8.3E-05			
T	TBXT, T-box transcription factor. ***Other aliases***: T brachyury homolog; LOC292301.	Encodes DNA-binding transcription factor protein; involved with cellular response to retinoic acid. Associated with neural tube defects and chordoma	cg17188046	0.111	0.551	0.440	4.3E-05	0.252	0.623	0.371	1.1E-04	No information on methylation and T.	TSS200	Hypermethylated
			cg14638883	0.107	0.520	0.413	1.8E-05	0.276	0.534	0.257	1.6E-03			
			cg19675288	0.056	0.538	0.482	2.2E-04	0.226	0.612	0.386	9.5E-05			
			cg06073449	0.016	0.427	0.410	1.8E-03	0.158	0.615	0.457	4.2E-04			
			cg06463958	0.039	0.589	0.550	4.2E-04	0.241	0.667	0.426	1.4E-04			
WDR17	WD repeat domain 17	Plays a functional role in early stages of retinal development. Frequently altered in T cell malignancies.	cg11923920	0.148	0.629	0.481	3.8E-07	0.237	0.487	0.251	1.0E-03	Associated with Liver neoplasms, tobacco use and Autosomal recessive retinitis pigmentosa.	1stExon;5’UTR;1stExon;5’UTR	Hypermethylated
			cg08095852	0.039	0.591	0.552	4.3E-07	0.161	0.507	0.346	4.1E-04			
			cg27486637	0.027	0.570	0.543	4.4E-07	0.210	0.581	0.371	1.1E-03			
			cg08684639	0.039	0.577	0.537	2.2E-04	0.118	0.547	0.429	2.0E-05			
ZIK1	Zinc finger protein interacting with K protein 1	Basic function unknown	cg00800512	0.105	0.562	0.456	1.5E-05	0.248	0.520	0.272	1.1E-04	Methylated biomarker for esophageal SCC.	5’UTR; 1stExon; TSS200	Hypermethylated
			cg12060744	0.199	0.691	0.492	4.3E-06	0.297	0.713	0.416	3.1E-04			
			cg01046104	0.084	0.644	0.560	1.9E-07	0.322	0.697	0.374	6.1E-04			
			cg18579862	0.023	0.627	0.604	3.8E-07	0.199	0.661	0.462	3.5E-04			
			cg26246807	0.033	0.574	0.541	1.9E-04	0.245	0.675	0.430	6.2E-04			
ZNF154	zinc finger protein 154. ***Other aliases*:** KIAA2003, PHZ-92	Involved in transcriptional regulation	cg03142586	0.033	0.504	0.471	1.2E-03	0.200	0.444	0.244	1.2E-03	Hypermethylation in 15 of 16 distinct cancer types from TCGA	Body; 5’UTR; 1stExon; TSS200	Hypermethylated
			cg11294513	0.126	0.576	0.450	1.2E-03	0.310	0.605	0.295	1.1E-03			
			cg05661282	0.055	0.595	0.540	7.1E-04	0.222	0.610	0.389	4.5E-04			
			cg21790626	0.030	0.523	0.494	9.1E-04	0.160	0.481	0.321	3.4E-04			
			cg27049766	0.088	0.576	0.489	8.2E-04	0.273	0.636	0.363	4.2E-04			
			cg03234186	0.134	0.533	0.399	1.7E-03	0.262	0.589	0.327	4.3E-04			
			cg08668790	0.133	0.583	0.450	1.3E-03	0.291	0.631	0.340	1.2E-04			
			cg12506930	0.163	0.560	0.397	1.0E-03	0.324	0.637	0.313	3.8E-04			
ZNF177	Zinc finger protein 177	Involved in transcriptional regulation	cg05928342	0.087	0.466	0.380	4.1E-05	0.240	0.550	0.310	4.3E-05	Methylated in gastric and hepatocellular cancer. Promoter methylation frequent in Endometrial cancer.	TSS200	Hypermethylated
			cg13703871	0.112	0.670	0.558	2.7E-07	0.300	0.671	0.371	6.7E-05			
			cg08065231	0.071	0.613	0.541	5.6E-08	0.299	0.646	0.347	2.5E-04			
			cg09578475	0.153	0.730	0.577	2.2E-08	0.456	0.724	0.268	8.1E-04			
			cg07788092	0.171	0.625	0.453	5.5E-08	0.384	0.637	0.253	1.7E-04			
ZNF529 / ZNF382	Zinc finger protein 529 a.k.a. KIAA1615	May be involved in transcriptional regulation. Molecular Function: DNA binding	cg25397945	0.039	0.595	0.556	1.0E-06	0.238	0.537	0.299	2.4E-03	No information on methylation and ZNF529.	Body; 1stExon; 5’UTR; TSS200; 1stExon; 5’UTR	Hypermethylated
			cg02587316	0.050	0.580	0.530	2.4E-05	0.200	0.522	0.322	5.0E-04			
			cg18630667	0.048	0.606	0.558	1.0E-04	0.176	0.492	0.316	3.7E-04			
			cg05020604	0.036	0.586	0.550	7.6E-05	0.195	0.504	0.310	1.9E-04			

**Fig 2:**
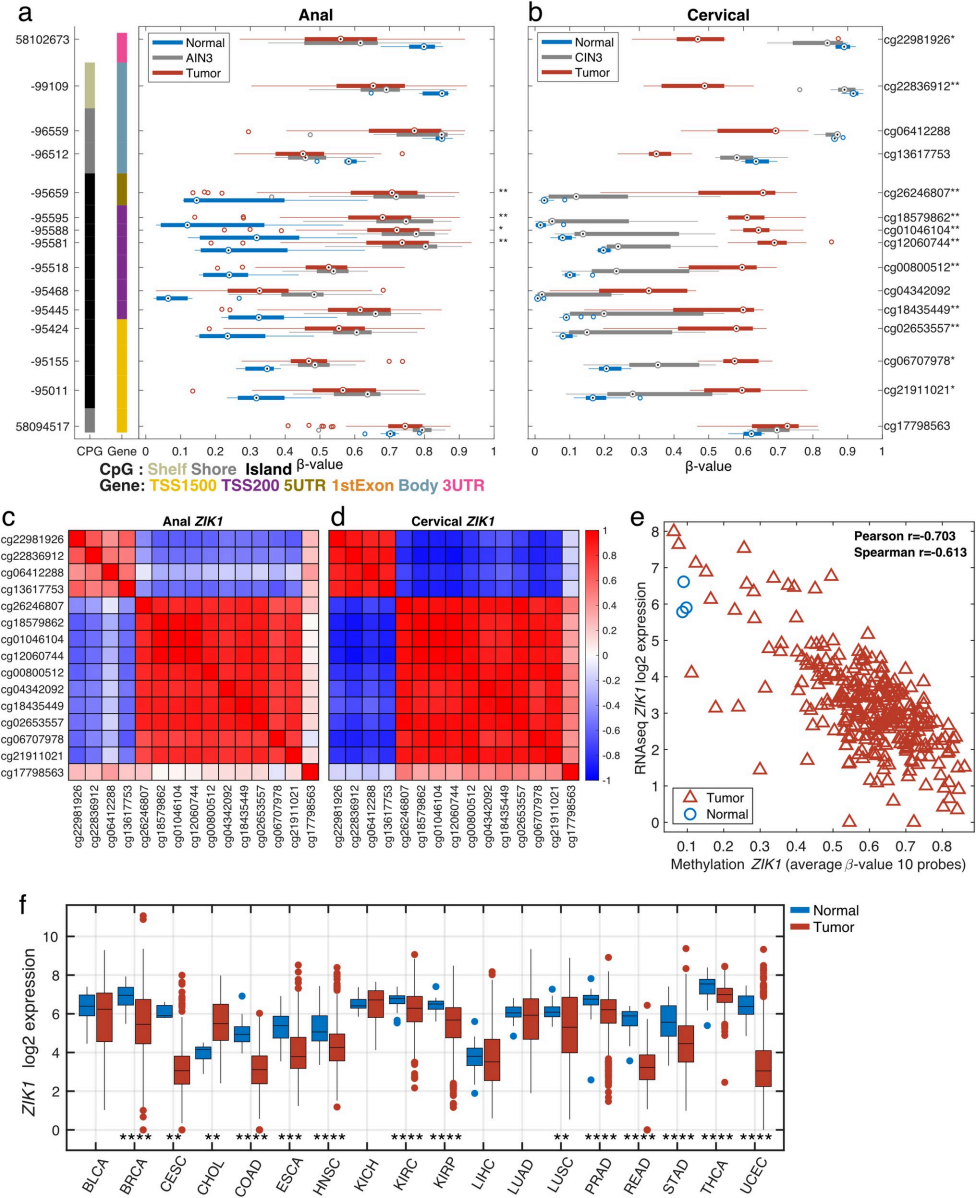
Gene structure methylation plot of differentially methylated CpG loci within *ZIK1* in anal (a) and cervical (b) tissues. Genomic coordinates are represented on the left vertical axis and methylation probe IDs (or CpG loci) on the right vertical axis. For each of the 15 CpG loci, boxplots illustrate the median (dot) and interquartile ranges [25th (low boundary of box) and 75th (upper boundary of box) percentiles] of β-values in tumor (red boxes), AIN3 or CIN3 (grey boxes) and normal (blue boxes). Significantly different median methylation at each CpG loci is noted as * for p<0.05 and ** for p<0.005. Among the 15 CpG sites presented, 4 CpG sites fall within the overlapping DMRs that were significantly hypermethylated in both anal (**a**) and cervical (**b**) cancers. For *ZIK1*, the anal in situ (AIN3) samples showed similar methylation levels to those of tumor samples whereas for CIN3, methylation levels were similar to normal cervical tissues. Corresponding correlation plots between all *ZIK1* probes for anal (**c**) and cervical (**d**) cancers show a high degree of correlation for ten of the probes. The average methylation levels for the ten correlated probes show high correlation (r = -0.7) to RNAseq gene expression levels in the TCGA CESC dataset (**e**). The tumor vs. normal expression levels across multiple TCGA tumor types are shown (**f,** *p<0.05, **p<0.01, ***p<0.001 & ****p<0.0001).

Zinc finger protein interacting with K protein 1 (*ZIK1*), a representative significant differentially methylated gene common to both anal and cervical analyses, is visualized in **Fig 2**, across multiple datasets. **Fig 2A and 2B** shows methylation levels for 15 CpG loci for each sample type in the anal (**a**) and cervical (**b**) datasets. In both anal and cervical tissues, many of the loci displayed hypermethylation in the tumor (red) samples compared to the normal (blue) samples. The AIN3 (grey) samples were hypermethylated (**a**) similar to the tumor samples while the CIN3 (grey) samples for cervical had a lower degree of methylation (b). Ten of the loci located in the transcriptional start site (TSS)-1500, TSS-200 and 5’UTR regions were highly correlated to each other in both the anal and cervical datasets (**Fig 2C and 2D**). **Fig 2E** shows the correlation between the average methylation level for the 10 highly correlated loci and RNAseq gene expression level for *ZIK1* in the TCGA CC dataset. The expression of *ZIK1* was observed to be lower in many of the TCGA tumor types (**Fig 2F)**.

**Fig 3:**
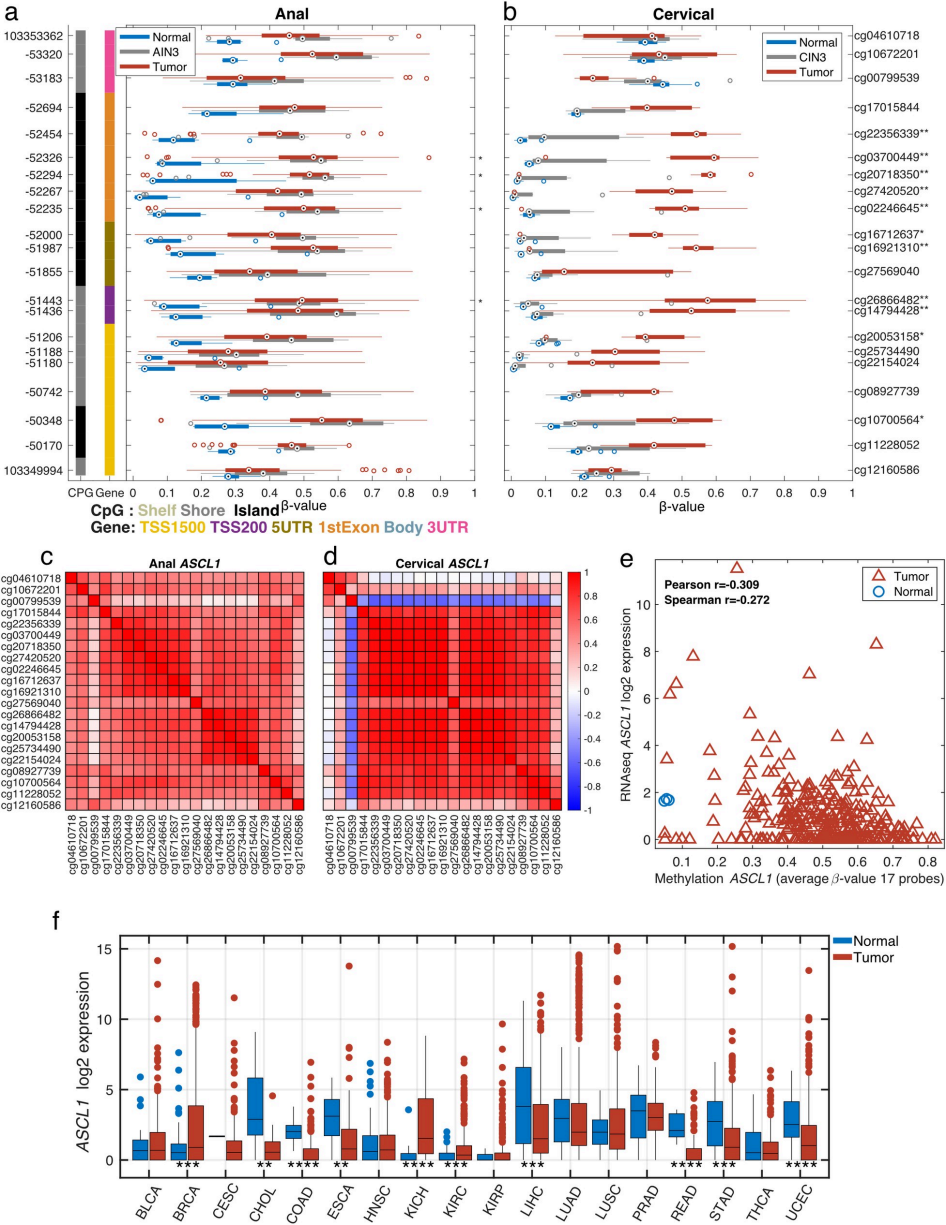
Gene structure methylation plot of differentially methylated CpG loci within *ASCL1* in anal (a) and cervical (b) tissues. Genomic coordinates are represented on the left vertical axis and methylation probe ID (or CpG loci) on the right vertical axis. For each of the 21 CpG loci, boxplots illustrate the median (dot) and interquartile ranges [25th (low boundary of box) and 75th (upper boundary of box) percentiles] of β-values in tumor (red boxes), AIN3 or CIN3 (blue boxes) and normal (green boxes). Significantly different median methylation at each CpG loci is noted as * for p<0.05 and ** for p<0.005. Among the 21 CpG sites presented, 4 CpG sites fall within the overlapping DMRs that were significantly hypermethylated in both anal (**a**) and cervical (**b**) cancers. For *ASCL1*, anal *in situ* (AIN3) showed similar methylation levels as tumors while, CIN3 methylation levels were similar to normal cervical tissues. Corresponding correlation plots between all *ASCL1* probes for anal (**c**) and cervical (**d**) cancers show a high degree of correlation for all of the probes. The average methylation levels for 17 probes show low correlation (r = -0.3) to RNAseq gene expression level in the TCGA CESC dataset (**e**). The tumor vs. normal gene expression levels across multiple TCGA tumor types are shown (**f**, *p<0.05, **p<0.01, ***p<0.001 & ****p<0.0001).

**Fig 3** provides a representative plot of a genomic region within the Achaete-scute family bHLH transcription factor 1 *(ASCL1*) gene. **Fig 3A** presents box plots of the methylation β-values in normal (blue), AIN3 or CIN3 (grey) and tumors (red) across 21 CpG sites. Among the 21 CpG sites presented, 4 CpG sites fall within the overlapping DMRs that were significantly hypermethylated in both AC (**Fig 3A**) and CC (**Fig 3B**). Methylation levels in AIN3 samples were more aligned with AC samples, whereas methylation levels in CIN3 were similar to those of normal cervical tissues. Corresponding plots between all *ASCL1* probes for ACs and CCs demonstrate a high degree of correlation (**Fig 3C and 3D)**. The pattern of the few probes with lower correlation is also similar between the anal and cervical data. The correlation between methylation and gene expression levels is lower for *ASCL1* and it is noteworthy that many samples show low mRNA expression of *ASCL1* in CCs from TCGA (**Fig 3E)**. Low expression is observed across most tumor types in TCGA (**Fig 3F)**. Similar patterns of hypermethylation in both anal and cervical cancers were observed for all 17 overlapping genes (**Table 1**).

### Derivation of AC and CC Partial Least Squares (PLS) methylation models

#### PLS scoring model differentiates AC from normal anal mucosae

**Fig 4:**
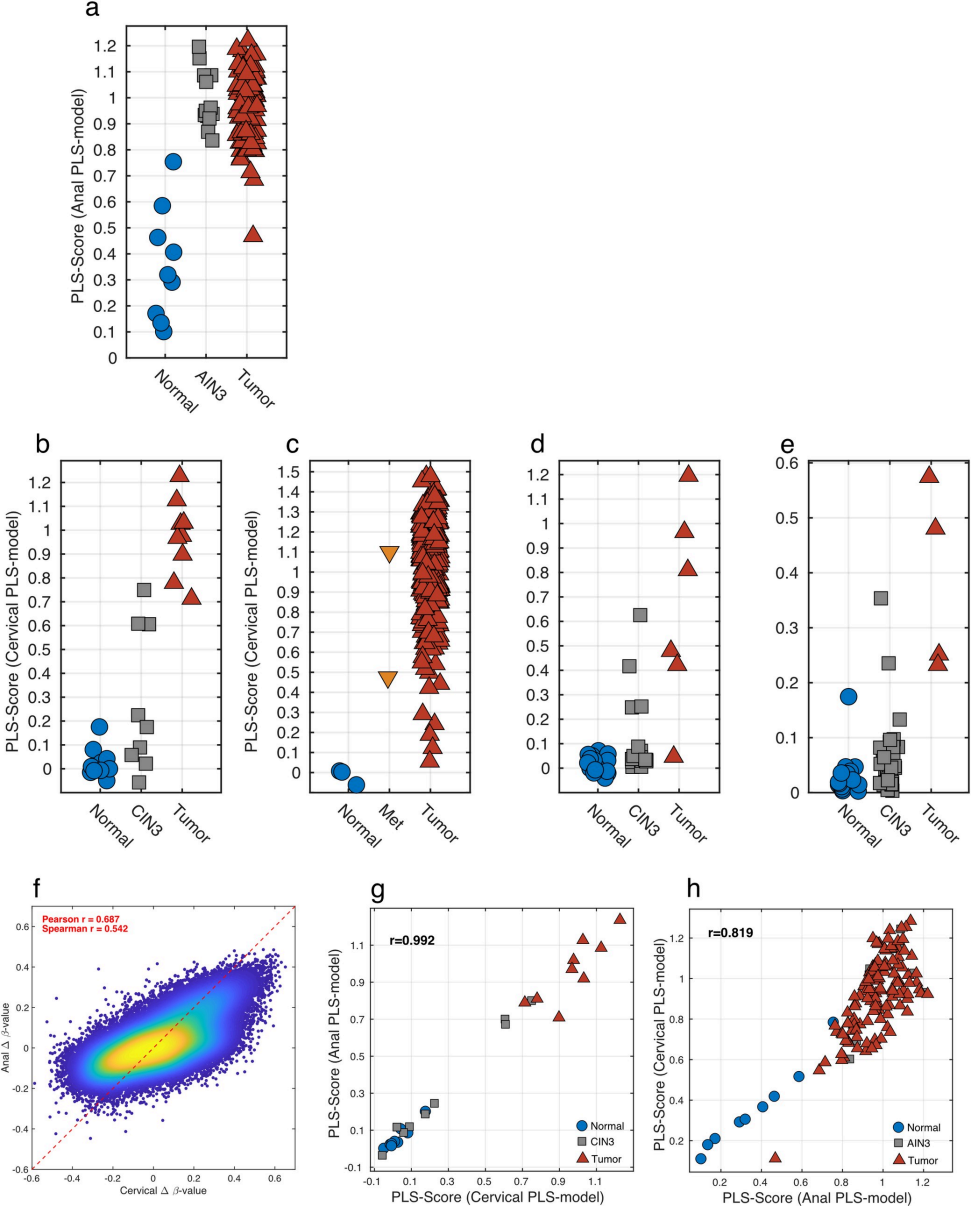
Methylation models for Anal (a-b) and Cervical (c-f) Cancer progression and similarities between Anal and Cervical methylation (g-h). The AC progression PLS model applied to the Anal dataset showed a clear distinction of normal and tumor samples, with the AIN3 samples scoring as tumor like (a). The CC progression PLS model applied to the Cervical dataset differentiates normal and the tumor samples with 3 of the CIN3 samples scoring as tumor-like and 6 as normal-like (b). The CC progression PLS model was further validated on three additional datasets. The TCGA Cervical dataset where the normal (n = 3) samples scored low, the metastatic (Met, n = 2) samples scores as tumors and all but 5 tumor (n = 307) samples scored high (c). In the GSE46306 cervical dataset, all of the normal (n = 20) samples scored as “normal” and most of the tumor (n = 6) scored as tumors while most of the CIN3 (n = 18) scored as “normal-like” with several being classified as “tumor-like” (d). Finally, in GSE99511 all normal cases (n = 28) scored appropriately while tumors (n = 4) scored higher with the majority but not all CIN3 cases (n = 36) scoring as “normal-like” (e). Density scatter plot for Δ β-values for tumor versus normal for cervical tissues on the x-axis and anal tissues on the y-axis (f). The high correlation indicates that the Δ β-values are similar when comparing the progression of both cervical and anal cancers. This was further explored by applying the AC progression PLS model to the cervical dataset and comparing it with the CC progression PLS model (g). The high correlation implies that the methylation changes are similar in cervical between anal cancers. This was further corroborated, when the CC progression PLS model was applied to the anal dataset and a similar high correlation was observed (h).

An AC progression PLS methylation model was derived using the selected DMRs (355 probes) and 130 samples (121 tumors, 9 normal samples) with a binary response. Cross-validation indicated that 2 PLS components were optimal. The PLS model explained 67% of the variation in X and 62% of the variation in Y (47% cross-validated). **Fig 4A** shows the calculated Y-values, or PLS-Score, for the normal anal and tumor samples and also the predicted Y-values for AIN3 samples. Sensitivity and specificity values for tumor vs. normal anal tissue were 0.99 and 0.78, respectively. PLS regression modeling was applied to all 143 anal tissue samples and a distinct separation was observed between the normal tissues when compared to both AIN3 and tumor samples (**Fig 4A**). All AIN3 samples were noted to segregate with the AC samples, which is consistent with the fact that all AIN3 were adjacent to an invasive AC.

#### PLS scoring model differentiates CC from normal epithelium

A PLS scoring model for CC was derived using the selected DMRs (172 probes) and 19 samples (9 tumors, 10 normal samples) with a binary response. Cross-validation indicated that one PLS component was optimal. The PLS model explained 93% of the variation in X and 95% of the variation in Y (94% cross-validated). The CC progression PLS methylation model had the ability to distinguish between normal and cancer samples with both sensitivity and specificity values being 1.0, as evidenced by the separation in calculated Y-values, or PLS-Score, for normal and CC samples (**Fig 4B)**. When the model was applied to the 9 CIN3 samples, predicted Y-values for CIN3 were observed to segregate into separate normal-like and tumor-like cases (**Fig 4B**).

### Validation of the CC progression PLS methylation model

The CC progression PLS methylation model was validated in 3 independent publicly available datasets. Firstly, we applied the model to HM450 BeadChip methylation data from 312 CC samples in The Cancer Genome Atlas (TCGA) [25]. Despite a small number of normal cervix samples, the cervical progression PLS methylation model demonstrated a robust ability to segregate normal cervix from CCs, with all but 8 cervical tumors accurately classified (false-negative rate of <3%) and all normal samples correctly classified (**Fig 4C**). Sensitivity and specificity values for tumor vs. normal in the TCGA dataset were 0.97 and 1.0, respectively.

Secondly, the CC progression PLS methylation model was applied to HM450 data obtained from 20 normal cervical tissues, 17 CIN3 and 6 CC samples and deposited within GEO (GSE46306) [13] The PLS model performed similarly to data obtained in this study as the predicted PLS score was able to segregate normal from CC specimens with sensitivity and specificity values of 0.5 and 1.0, respectively (**Fig 4D**). Furthermore, the PLS model clustered the majority of CIN3 samples with the normal cervical samples; however, three CIN3 lesions clustered with cervical tumors and may be classified as having “tumor-like” or high-risk methylation patterns.

Finally, our model was applied to the HM450 from Verlaat et al [18] with 28 normal, 36 CIN3 and for 4 tumor sample (**Fig 4E)**. The PLS showed a compressed range with the highest tumor scoring 0.6; however, there was still a clear separation between normal and tumor samples, with most of the CIN3 samples scoring as normal-like. For this (GSE99511) dataset, we obtained a sensitivity of 0.25 and specificity of 1.0 when comparing normal vs. tumor samples.

In summary, analysis of CIN3 lesions using the PLS model, effectively groups high grade cervical dysplasia into subsets of CIN3 specimens that display DNA methylation patterns similar to either normal cervical tissue or invasive CC.

### Cross-application of cervical and AC progression PLS methylation models

Given that there were 17 differentially methylated genes that overlapped between the cervical and ACs, and that the Δ β-values were similar in both the anal and cervical datasets (**Fig 4F**), the cross-applicability of the two PLS methylation models was examined. When the AC progression PLS methylation model, which included all 355 probes, was applied to the cervical methylation data, a high correlation with the CC progression PLS model was observed that included 173 CpG probes (**Fig 4G**). Similarly, the converse application of the CC progression PLS methylation model to the anal methylation data yielded a similar high correlation (**Fig 4H**).

## Discussion

HPV-associated cancers remain an important health issue in the US with the incidence of AC continuing to rise and CC and oropharyngeal cancer comprising the largest proportion of such malignancies [1]. Unlike for AC, screening and prevention guidelines for CC are well developed and have been successful in reducing the number of new cases [29]. However, given the high prevalence of HSIL/CIN2 and an inability to predict regression of CIN2/3 lesions, it can be presumed that a substantial number of women may be overtreated with excision or ablation.

Although there are similarities in the underlying carcinogenesis, AC screening standards remain less evolved than those for CC [6]. It has been suggested that high-risk populations such as HIV+ individuals, MSM, and immunosuppressed patients should be considered for AC screening [30]. However, the adaptation of cervical screening to the anus, such as High Resolution Anoscopy (HRA) [31] following abnormal anal cytology has limitations due to differences in anatomic site that impact the screening procedure [6, 32]. These limitations were highlighted in a study of 138 HIV+ MSM diagnosed with AC; of whom over half had participated in HRA-based screening prior to diagnosis and a significant number of ACs identified were located where a preceding AIN3 was treated [33]. Similar to CC, there is also a concern regarding overtreatment by aggressive use of HRA and ablation for AIN3/HSIL, especially among high-risk populations. Although AIN3/HSIL lesions are potential precursors for malignant transformation, it is clear that the majority of such lesions actually do not progress to invasive disease [34]. The AC HSIL Outcomes Research (ANCHOR) study, that initiated patient accrual in 2014, is a randomized screening trial that may provide insight into the benefit of identification and treatment of HSIL through screening and HRI as it relates to the progression to HSIL to malignancy [35, 36]. Ongoing efforts to better refine screening will be needed to supplement the findings of ANCHOR.

Molecular biomarkers for identifying HPV-associated pre-cancerous lesions at high risk for progression to malignancy would be valuable for enhanced screening, targeted prevention and to reduce overtreatment. Approaches to identify epigenetic alterations in HPV-associated cervical and anal cancer have evolved from a targeted approach of known epigenetic targets in cancer to larger panels of targeted genes. Early work focused on panels of small numbers of selected cancer specific genes, such as APC, CALCA, CNNA1, C13ORF18, DAPK1, ESR1, RARB, SLIT2 or WIF1, to differentiate normal cervix from CC [37–39]. Subsequent work using a high throughput qMSP-based targeted approach, the investigators from the University of Groningen examined a targeted panel of 213 cancer-specific genes and derived a 4-gene panel (JAM3, EPB41L3 and TERT and C13ORF18) that was able to discriminate CIN3 and CC more accurately than conventional cytology [40]. Lendvai et al. [41] used Methylated DNA Immunoprecipitation (MeDIP) combined with DNA microarray to identify two differentially methylated regions of COL25A1 and KATNAL2 genes as having significantly progressive methylation with increasing severity of CIN compared with normal cervical epithelium. Using the MethylCap-Seq platform for genome-wide methylation analysis, Boers et al. identified 8 new candidate methylated markers that distinguished CIN2/3 from normal cervix (ZSCAN1, ST6GALNAC5, ANKRD18CP, CDH6, GFRA1, GATA4, KCNIP4, and LHX8) [42]. When combined with their previously identified 4 gene panel, C13ORF18, JAM3 and ANKRD18CP were the best discriminatory combination of methylated genes for detection of high-grade CIN [42, 43]. Verlaat et al. applied the technique of next-generation sequencing of methyl-binding enriched DNA (MBD-Seq) and identified 3 methylated genes (GHSR, SST and ZIC1) that in combination with 3q chromosomal gain showed high rate of detection of high-grade CIN [17]. Subsequently, that same group applied the HM450 platform to identify differential methylation between normal and CIN3 tissues (and no cervical cancer) which yielded 12 candidate markers which were then narrowed down to a 3-gene classifier (ASCL1, LHX8 and ST6GALNAC5) for the detection of CIN3 in high-risk HPV+ self-samples [18].

In this study, we used stringent analytical methodology that reduced genome-wide HM450 epigenetic data into a common 17 gene epigenetic classifier for CC and AC. Similar to our approach, Farkas et al. were among the first to apply the same HM450 platform for a comparison of differential methylation between 6 cervical cancers, 18 CIN3 and 20 normal cases [13]. They identified 6 genes as the best candidate methylated biomarkers of cervical cancer progression (RGS7, LHX8, ST6GALNAC5, TBX20, KCNA3 and ZSCAN18). Both this report and that of Farkas [13] identified targets of differential methylation between cancer and normal tissue and examined if those DMR were able to identify higher risk high-grade cervical and anal neoplasia. This is an alternative yet complementary approach to work published by the University of Groningen group which focused only on differentiating CIN2/3/HSIL from normal cells. Our joint CC and AC classifier, and the cancer-specific classifiers reported herein, independently identified DMR in genes that have previously been identified as being epigenetically altered in CIN2/3 mostly by targeted panel approaches. The most prominent of these include overlap of ASCL1 and WDR17 [12, 18] in our joint model and, ZNF582, ST6GALNAC5, and c13orf18 in the disease-specific models [13, 15, 18, 42]. Of note, our joint classifier contains ASCL1, which is one of the three markers identified by Verlaat et al. using HM450 arrays to differentiate CIN3 in high-risk HPV+ self-samples [18] and was also reported as an epigenetic marker in a gene panel developed for detection of oral SCC, another HPV-associated cancer [44]. Hypermethylation of *PAX1* has been reported as a candidate methylated biomarker for oral dysplasia/cancer detection and used to differentiate normal cervical mucosa from CC specimens [45]. Such shared methylation changes suggest that HPV induces a number of non-random changes in the host methylome that may in turn, contribute to carcinogenesis. As noted, such alterations have the potential to be leveraged as clinical biomarkers and/or therapeutic targets [46].

The identification of shared differentially methylated targets leading to mutually applicable progression signatures between cervical and anal squamous carcinogenesis is a significant strength of this study. Given that the methylation signatures were derived from completely different sources, were preserved differently (fresh frozen vs. FFPE), processed and analyzed by methylation arrays at different times, it is highly compelling that they ultimately yielded a remarkable number of shared methylation targets as well as interchangeable cancer progression signatures. This certainly speaks to the robustness of the PLS methylation models. Furthermore, the CC progression PLS model was validated using three independent datasets [12]. In addition, we were the first group to apply the HM450 platform for the analysis of invasive AC [11] and to our knowledge, this study is the first to report the comprehensive application of HM450 to a patient sample set to directly derive a progression signature for anal neoplastic progression. In summary, using a comprehensive approach, we built and developed a PLS model-based classifier in CC, cross-validated the classifier using external open-source data and then, based on the high correlation between methylation status in CC and AC, we integrated the CC and AC models to develop a joint PLS classifier.

The shared CC and AC PLS methylation model (differentiating normal tissue from cancer) was able to segregate CIN3 or AIN3 cases into normal-like and cancer-like groups. The application of this model has potential implications for risk stratification of high-grade lesions. This concept of using methylated genes to define cancer risk heterogeneity among CIN2/CIN3 lesions was also described by Verlaat et al. [18, 47]. Interestingly, by arbitrary application of a similar panel of methylation markers of cervical neoplastic progression to anal cancer progression, van der Zee et al. have demonstrated the detectability of AIN3 in both HIV+ and HIV- patients but also the ability to define potential patterns of higher cancer risk in these high-grade lesions [12, 19, 48].

As part of our shared AC/CC classifier, we identified a number of genes that are known to play a role in malignancies or key cancer-related cellular functions such as cell cycle regulation and cell adhesion. *SORCS3*, *ZNF154* and *ZNF*177 are hypermethylated in gastric cancers [49–51], while ZNF177 is also hypermethylated in hepatocellular carcinomas [52]. Frequent methylation of MIR129-2 has been observed in both lymphoid malignancies and during the progression of monoclonal gammopathy of unknown significance (MGUS) to multiple myeloma [53]. In T-cell malignancies, recurrent missense and nonsense mutations have been identified in *WDR17* [54]. *FMN2* regulates cell cycle progression from G1 to S phase by regulation at p21 [55] and modulates adhesion stability via actin bundle regulation [56]. With respect to other genes, *ZIK1* is a transcriptional repressor [57], *ASCL1* induces tight junction protein Cldn5 [58] and *MARCH11* is expressed in the testes and enhances lysosomal degradation and delivery [59].

Our study has certain inherent limitations. First, the CC progression PLS model was derived from a small sample set; however, the model was validated in three larger CC datasets. Second, the AC progression PLS model was generated in a retrospective analysis; however, the source of the AC patient population and tissues was part of a prospective national trial with systematic data and tissue collection methodology. The AC and CC specimens differed by preservation method (FFPE vs. fresh frozen, respectively); therefore, a combined PLS model could not be generated. In addition, the number of analyzable non-cancer anal tissue samples was small but reflected the inherent rarity of co-existing normal, AIN3 and invasive cancer samples from a common patient population. Although the AIN3 specimens were derived from tissue adjacent to or in association with invasive cancer, we would emphasize that both the AIN3 and normal tissues were frequently derived from separate paraffin blocks and if they were from the same section, they were clearly spatially delineated with confirmation by 2 independent pathologists with subsequent meticulous microdissection. It is acknowledged that there could be a possible field effect of similar methylation patterns (or contamination) adjacent to invasive cancer. The fact that adjacent normal tissues were distinguishable from tumor across 355 significant CpG loci representing 84 significant genes supports the fact that this PLS model is not overly influenced by invasive tumor field effect or contamination. However, the similarities between AIN3 and AC may be higher than that of normal tissue and may have led to the co-segregation of AIN3 with invasive ACs. Notably, in the cross-application of AC to CC, the AC signature was able to independently dichotomize CIN3 cases into normal-like and cancer-like specimens and provides additional reassurance.

It is well known that both cervical and anal mucosa when infected by HPV, follow a predictable progression from intraepithelial neoplasia to invasive malignancy. Due to the current inability to specifically distinguish high grade lesions that will progress to malignancy, ablative procedures are routinely recommended on all such lesions for the prevention of AC and CC. The identification of segregating biomarkers to better select those high-grade lesions which should be treated or observed would have a high clinical impact. It has been shown that there are distinct and shared methylation changes that occur across the genome during the progression of AC and CC. The profile of epigenetic alterations between these two cancer types is highly similar, suggesting that HPV-driven oncogenesis may result in similar non-random methylomic events. Herein, we identified shared epigenetic alternations between AA and CC and developed an integrated joint PLS methylation model for both CC and AC. This has implications for the future development of shared biomarkers as well as epigenotype-phenotype associations. Larger-scale validation studies and evaluation in other HPV-associated malignancies are warranted.

## Supporting information

S1 Fig **a-b.** β-value histograms for anal and cervical tissues, respectively. The β-value histograms for the anal dataset (**a**) all show a bimodal distribution, with some tumor samples (red-line) demonstrating a third peak and some degradation observed. A similar trend was observed for the cervical dataset (**b**) but without degradation. This is likely attributable to the fact that the anal specimens were FFPE while the cervical samples were fresh frozen.
(TIF)

S2 Fig **a-b.** t-SNE was used to cluster the samples and separated normal (blue circles) from the tumor (red triangles) samples. AIN3 (grey squares) samples tended to be interspersed among the tumor samples in the anal dataset (**a**), while CIN3 (grey squares) cases segregated more closely to normal cervical tissues (**b**).
(TIF)

S1 Table Patient demographics for cervical and anal cancer cases. (DOCX)

S2 Table Anal cancer genes. The comparison of normal anal mucosae with ACs yielded 355 differentially methylated CpG loci representing 86 discrete genes.
(DOCX)

S3 Table Cervical cancer genes. By DMR-defining criteria, 36 separate genes comprised of 173 CpG loci that significantly distinguished CC from normal cervical tissue were identified.
(DOCX)
