# Supplementary figures and images for: Genome-wide host methylation profiling of anal and cervical carcinoma
Source: PLoS One. 2021 Dec 9;16(12):e0260857. doi: 10.1371/journal.pone.0260857 (PMC8659695; doi:10.1371/journal.pone.0260857)

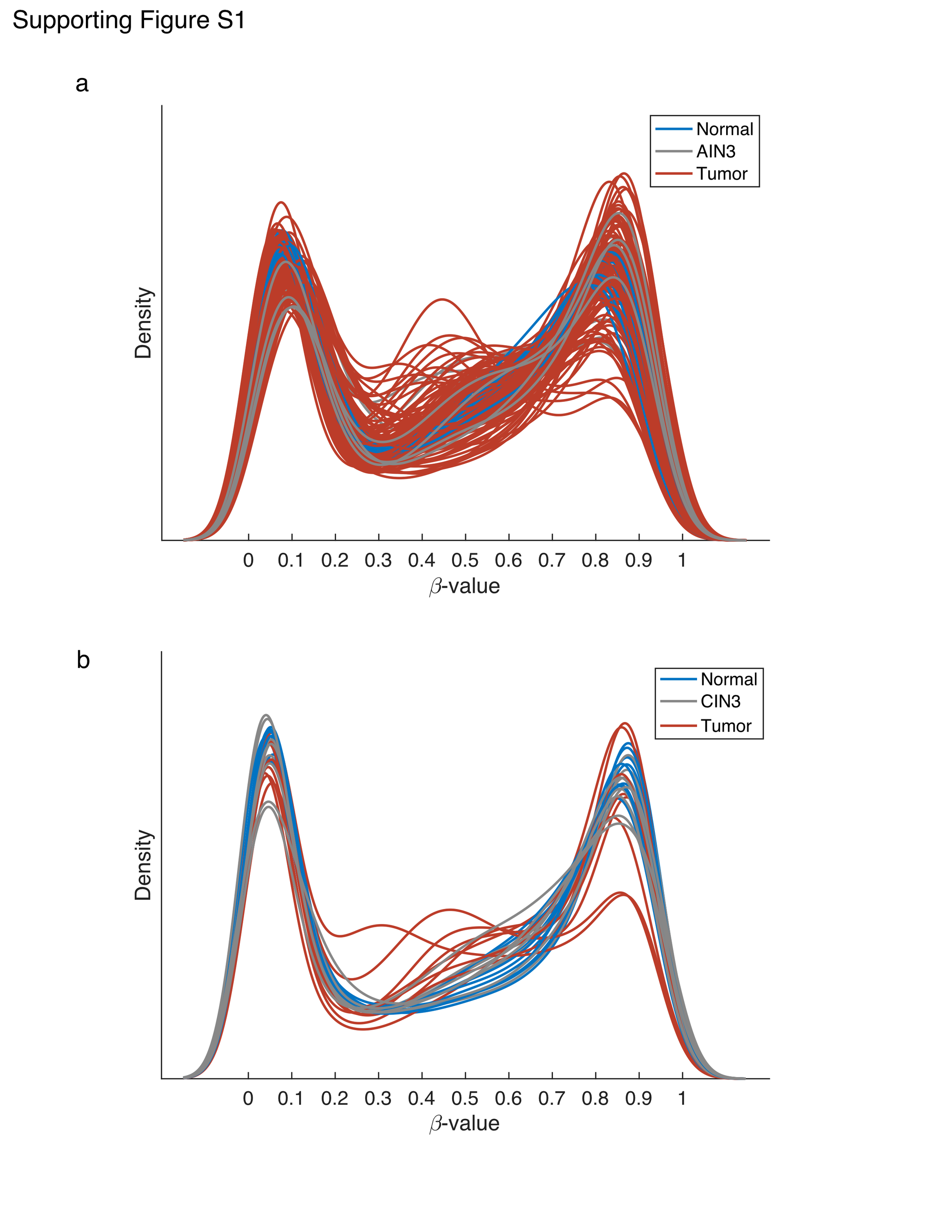

Supplement: S1 Fig — a-b. β-value histograms for anal and cervical tissues, respectively. The β-value histograms for the anal dataset (a) all show a bimodal distribution, with some tumor samples (red-line) demonstrating a third peak and some degradation observed. A similar trend was observed for the cervical dataset (b) but without degradation. This is likely attributable to the fact that the anal specimens were FFPE while the cervical samples were fresh frozen. (TIF) [file pone.0260857.s001.tif]

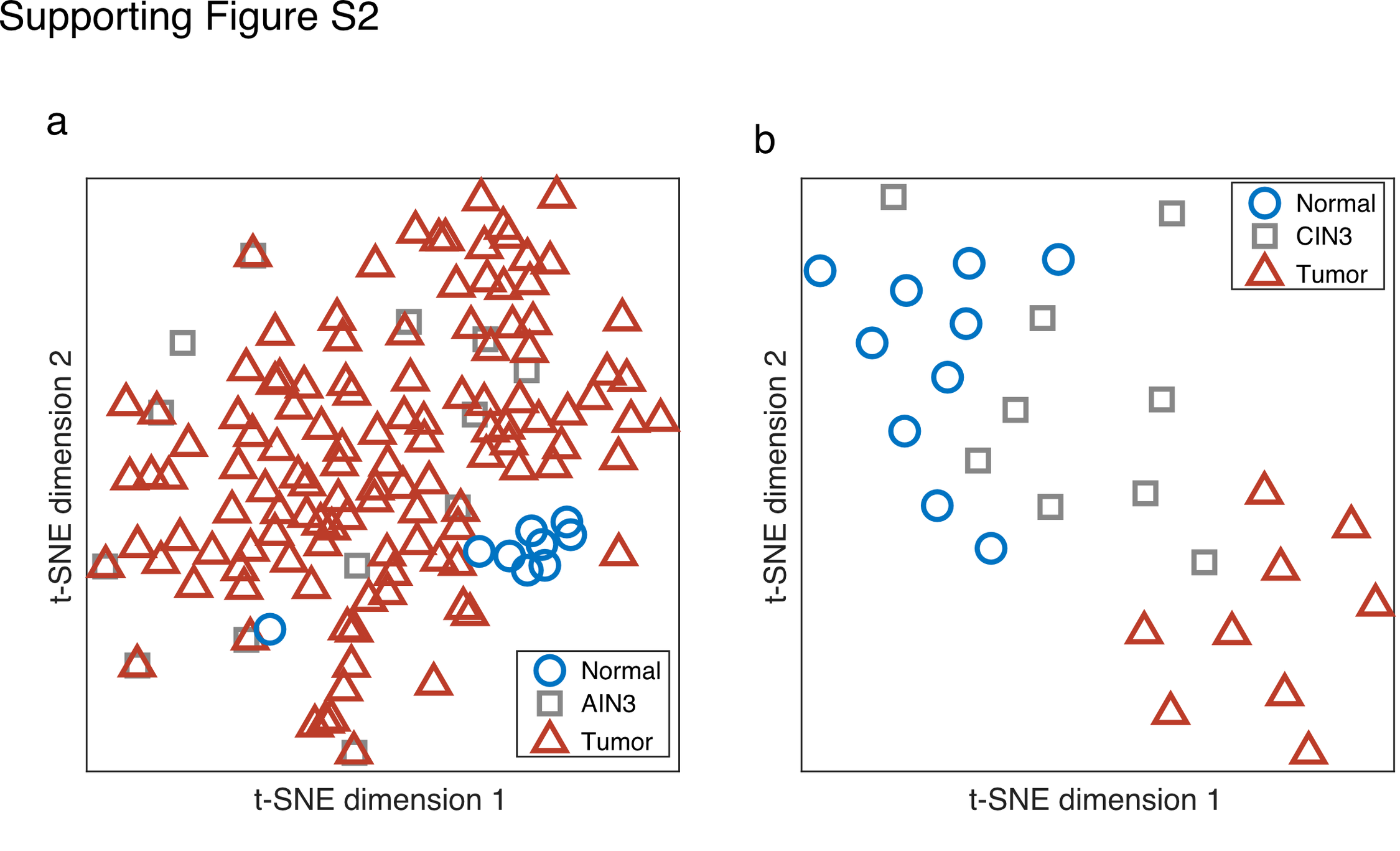

Supplement: S2 Fig — a-b. t-SNE was used to cluster the samples and separated normal (blue circles) from the tumor (red triangles) samples. AIN3 (grey squares) samples tended to be interspersed among the tumor samples in the anal dataset (a), while CIN3 (grey squares) cases segregated more closely to normal cervical tissues (b). (TIF) [file pone.0260857.s002.tif]
