# Supplementary material for: Genome-wide host methylation profiling of anal and cervical carcinoma
Source: PLoS One. 2021 Dec 9;16(12):e0260857. doi: 10.1371/journal.pone.0260857 (PMC8659695; doi:10.1371/journal.pone.0260857)
Supplement: S1 Table — (DOCX) [file pone.0260857.s003.docx]

**S1 Table. Patient Demographics for Cervical and Anal Cancer Cases**

|  | **Total Cervical** | | **Total Anal Cases** | |
| --- | --- | --- | --- | --- |
|  | **n = 26** | | **n = 121** | |
|  | Overall | (%) | Overall | (%) |
| **n** | 26 | | 121 | |
|  |  | |  | |
| **Median Age** | 35 | | 54 | |
| (min-max) | (22-68) | | (25-79) | |
|  |  |  |  |  |
| **Race** |  |  |  |  |
| White | 21 | 81 | 105 | 87 |
| Black /African American/Other | 5 | 19 | 16 | 13 |
|  |  |  |  |  |
| **Gender** |  |  |  |  |
| Male | 0 | 0 | 47 | 39 |
| Female | 26 | 100 | 74 | 61 |

Abbreviations: min, minimum; max, maximum; SD, standard deviation
