# Supplementary material for: Genome-wide host methylation profiling of anal and cervical carcinoma
Source: PLoS One. 2021 Dec 9;16(12):e0260857. doi: 10.1371/journal.pone.0260857 (PMC8659695; doi:10.1371/journal.pone.0260857)
Supplement: S2 Table — The comparison of normal anal mucosae with ACs yielded 355 differentially methylated CpG loci representing 86 discrete genes. (DOCX) [file pone.0260857.s004.docx]

**S2 Table. Anal Cancer Genes**

| **Gene Symbol** | **Product** | **Function /Association with Disease** | **Aliases** | **Chr.** | **Position** | **CpG Number** | **Genomic Region** | **Island** |
| --- | --- | --- | --- | --- | --- | --- | --- | --- |
|  |  |  |  |  |  |  |  |  |
| ADCYAP1 | Adenylate Cyclase Activating Polypeptide 1 | Promotes neuron projection development through the RAPGEF2/Rap1/B-Raf/ERK pathway and is a key mediator of neuroendocrine stress responses. | Adenylate Cyclase Activating Polypeptide 1; Pituitary Adenylate Cyclase-Activating Polypeptide; Prepro-PACAP; PACAP | 18 | 904523 | cg19962990 | TSS1500 | chr18:904578-909574 |
|  |  |  |  |  | 904851 | cg16457786 | TSS1500  TSS200 |  |
|  |  |  |  |  | 904878 | cg24287438 |  |  |
|  |  |  |  |  | 904885 | cg21438101 |  |  |
| ASCL1 | Achaete-scute family bHLH transcription factor 1 | Among a panel of methylated genes for the detection of oral SCC. Methylated in anal cancer. | HASH1; Class A Basic Helix-Loop-Helix Protein 46, Achaete-Scute Homolog 1, BHLHa46, ASH-1, BHLHA46, MASH1 | 12 | 103352235 | cg02246645 | 1stExon | chr12:103351579-103352695 |
|  |  |  |  |  | 103352267 | cg27420520 | 1stExon |  |
|  |  |  |  |  | 103352294 | cg20718350 | 1stExon |  |
|  |  |  |  |  | 103352326 | cg03700449 | 1stExon |  |
| ATP10A | ATPase phospholipid transporting 10A (putative) | Possible imprinted gene. Methylated in colorectal cancer. | ATP10C, ATPVA, ATPVC, EC 3.6.3.1, EC 7.6.2.1, KIAA0566, EC 3.6.3, ATPase Type IV, Phospholipid Transporting (P-Type), Phospholipid-Transporting ATPase VA. | 15 | 26108391 | cg03419058 | TSS200 | chr15:26107503-26108818 |
|  |  |  |  |  | 26108399 | cg16389285 |  |  |
|  |  |  |  |  | 26108401 | cg20124450 |  |  |
|  |  |  |  |  | 26108410 | cg22113930 |  |  |
|  |  |  |  |  | 26108412 | cg26230285 |  |  |
| C13orf18 | Chromosome 13 Open Reading Frame 18 | Regulator of autophagy and may also function as a tumor suppressor. Associated with cervical disease and cervical cancer. | RUBCNL, Rubicon Like Autophagy Enhancer, RUN And Cysteine Rich Domain Containing Beclin 1 Interacting Protein Like. Protein Associated With KIAA0226L | 13 | 46961008 | cg15728861 | 5'UTR | chr13:46960684-46961670 |
|  |  |  |  |  | 46961115 | cg11854491 |  |  |
|  |  |  |  |  | 46961583 | cg23833588 | 1stExon;  5'UTR |  |
| C1orf114 | Chromosome 1 Open Reading Frame 114 | Functions in microtubule-binding protein that localizes to the microtubular manchette of elongating spermatids. Methylated and associated with Prostate cancer. | CCDC181, Coiled-Coil Domain-Containing Protein 181. | 1 | 169396635 | cg00100121 | 1stExon;  5'UTR | chr1:169396621-169396869 |
|  |  |  |  |  | 169396637 | cg13958426 |  |  |
|  |  |  |  |  | 169396706 | cg00002719 | TSS200 |  |
|  |  |  |  |  | 169396712 | cg08104202 |  |  |
|  |  |  |  |  | 169396834 | cg16998150 |  |  |
|  |  |  |  |  | 169396858 | cg08047907 |  |  |
|  |  |  |  |  | 169396868 | cg24808280 |  |  |
| CA3 | Carbonic Anhydrase 3 | CA3 is associated with acute Myocardial Infarction; hordeolum. | Carbonate dehydratase III; Carbonic anhydrase III; CA-III | 8 | 86350568 | cg05355225 | TSS1500 | chr8:86350765-86351196 |
|  |  |  |  |  | 86350581 | cg18674980 |  |  |
|  |  |  |  |  | 86350592 | cg07560510 |  |  |
| CALCA | Calcitonin Related Polypeptide Alpha | It induces vasodilation and dilates a variety of vessels including the coronary, cerebral and systemic vasculature. Associated diseases include malignant neoplas, migraine disorder, and hypertensive disease. | Calcitonin, Calcitonin Gene-Related Peptide 1, Calcitonin Gene-Related Peptide I, Alpha-Type CGRP, Calcitonin 1, CGRP-I, CALC1. | 11 | 14995138 | cg12083965 | TSS1500 | chr11:14995128-14995908 |
|  |  |  |  |  | 14995142 | cg11153872 |  |  |
|  |  |  |  |  | 14995167 | cg26676559 |  |  |
|  |  |  |  |  | 14995201 | cg22863523 |  |  |
|  |  |  |  |  | 14995231 | cg11697588 |  |  |
|  |  |  |  |  | 14995233 | cg16245265 |  |  |
| CCNA1 | Cyclin A1 | Functions primarily in the control of the germline. Associated with Liver carcinoma, leukemia, cervical cancer. | Cyclin A1 2 3 5  Cyclin-A1, Testicular Tissue Protein Li 34, CT146. | 13 | 37005562 | cg24334591 | TSS1500 | chr13:37005581-37006453 |
|  |  |  |  |  | 37005566 | cg00282249 |  |  |
|  |  |  |  |  | 37005570 | cg05089090 |  |  |
|  |  |  |  |  | 37005582 | cg08676975 |  |  |
|  |  |  |  |  | 37006063 | cg02478448 | 1stExon  5'UTR; |  |
|  |  |  |  |  | 37006107 | cg18348647 |  |  |
| CLDN10 | Claudin 10 | Plays a major role in tight junction-specific obliteration of the intercellular space, through calcium-independent cell-adhesion activity. Associated with liver carcinoma, neoplasm metastasis, and helix syndrome. | Oligodendrocyte-Specific Protein-Like, Claudin-10, OSP-Like Protein, OSP-Like, CPETRL3, HELIX. | 13 | 96204870 | cg04603730 | TSS200  Body | chr13:96204691-96205496 |
|  |  |  |  |  | 96204873 | cg10305311 |  |  |
|  |  |  |  |  | 96204917 | cg09469554 |  |  |
| CTNNA2 | Catenin alpha-2 | Regulates cell-cell adhesion and differentiation in the nervous system. Mutations in CTNNA2 is responsible for the disease, cortical dysplasia. | Alpha N-catenin, Alpha-catenin-related protein, CAPR, Alpha N-Catenin, CDCBM9, CAP-R, CT114, CTNR. | 2 | 80530255 | cg20072442 | Body | chr2:80529677-80530846 |
|  |  |  |  |  | 80530431 | cg24632241 |  |  |
|  |  |  |  |  | 80530701 | cg23665824 |  |  |
|  |  |  |  |  | 80530770 | cg17349389 |  |  |
|  |  |  |  |  |  |  |  |  |
| CTNND2 | Catenin Delta 2 | Has a critical role in neuronal development, particularly in the formation and/or maintenance of dendritic spines and synapses. CTNND2 is associated with schizophrenia, colorectal cancer, and unipolar depression. | Neurojungin, Catenin (Cadherin-Associated Protein), Delta 2 (Neural Plakophilin-Related Arm-Repeat Protein), Catenin Delta-2, NPRAP, GT24. | 5 | 11904110 | cg04996219 | TSS200 | chr5:11903550-11904703 |
|  |  |  |  |  | 11904114 | cg07195011 |  |  |
|  |  |  |  |  | 11904127 | cg13368756 |  |  |
| DCC | DCC Netrin 1 Receptor | The transmembrane protein is a member of the immunoglobulin superfamily of cell adhesion molecules, and mediates axon guidance of neuronal growth cones towards sources of netrin 1 ligand. The protein functions as a tumor suppressor, and is frequently mutated or downregulated in colorectal cancer and esophageal carcinoma. Also indicated in agenesis of corpus callosum. | Immunoglobulin Superfamily DCC Subclass Member 1, Tumor Suppressor Protein DCC, Netrin Receptor DCC, IGDCC1, Immunoglobulin Superfamily, DCC Subclass, Member 1, Putative Colorectal Tumor Suppressor, HGPPS2, NTN1R1, CRC18, CRCR1, MRMV1. | 18 | 49866335 | cg18801691 | TSS1500 | chr18:49868377-49868759 |
|  |  |  |  |  | 49866361 | cg19613722 | TSS200 |  |
|  |  |  |  |  | 49866371 | cg19042459 |  |  |
| DPP10 | Dipeptidyl Peptidase Like 10 | Methylated in nicotine-exposed fetal lung and placental tissue. May be associated with colorectal cancer. Seen in Autism, mood disorder or schizophrenia. | DPRP-3, DPP X, DPRP3, DPL2, KIAA1492, DPPY, Dipeptidyl-Peptidase 10. | 2 | 115919785 | cg04075191 | Body  1stExon  5'UTR | chr2:115918737-115920765 |
|  |  |  |  |  | 115919829 | cg01718116 | Body  1stExon |  |
|  |  |  |  |  | 115919950 | cg00089091 | Body |  |
|  |  |  |  |  | 115920221 | cg13777681 | Body |  |
| DPP6 | Dipeptidyl Peptidase Like 6 | DPP6 is a Protein Coding gene that promotes cell surface expression of potassium channel KCND2. It is associated with diseases such as; mental retardation and ventricular fibrillation. | Dipeptidyl Peptidase VI, Dipeptidyl Peptidase 6, DPP VI, DPPX, Dipeptidyl Aminopeptidase IV-Related Protein, Dipeptidyl Peptidase IV-Related Protein, Dipeptidyl Peptidase IV-Like Protein, Dipeptidylpeptidase VI, Dipeptidylpeptidase 6, MRD33, DPL1, VF2. | 7 | 153584582 | cg06495961 | 1stExon  5'UTR | chr7:153583317-153585666 |
|  |  |  |  |  | 153584597 | cg14523847 |  |  |
|  |  |  |  |  | 153584609 | cg27032232 |  |  |
|  |  |  |  |  | 153584748 | cg24011260 |  |  |
|  |  |  |  |  | 153584839 | cg03532926 | Body |  |
|  |  |  |  |  | 153584873 | cg14564076 |  |  |
|  |  |  |  |  |  |  |  |  |
| EDNRB | Endothelin Receptor Type B | EDNRB is a protein coding gene that encodes a G protein-coupled receptor responsible for activating a phosphatidylinositol-calcium second messenger system. Diseases associated with EDNRB include Hirschsprung Disease 2 and Waardenburg Syndrome, Type 4A. | Endothelin Receptor Non-Selective Type, ET-BR, ET-B, ETRB, Endothelin Receptor Subtype B1, ABCDS, HSCR2, ETB1, ETBR, WS4A, HSCR, ETB. | 13 | 78493205 | cg23766591 | TSS1500  5'UTR | chr13:78492425-78493382 |
|  |  |  |  |  | 78493215 | cg23702615 |  |  |
|  |  |  |  |  | 78493229 | cg18032190 |  |  |
|  |  |  |  |  | 78493297 | cg19650157 |  |  |
|  |  |  |  |  | 78493305 | cg13434989 |  |  |
|  |  |  |  |  | 78493313 | cg10016380 |  |  |
|  |  |  |  |  | 78493657 | cg02147695 |  |  |
|  |  |  |  |  | 78493666 | cg22541679 |  |  |
|  |  |  |  |  | 78493671 | cg23494140 |  |  |
| EPDR1 | Ependymin Related 1 | Upregulated In Colorectal Cancer. Protein coding gene that plays a role in calcium-dependent cell adhesion. | Mammalian Ependymin-Related Protein 1, MERP1, UCC1, Ependymin Related Protein 1 (Zebrafish), Mammalian Ependymin Related Protein 1, Ependymin Related Protein 1, EPDR | 7 | 37960873 | cg10876076 | Body | chr7:37960316-37961046 |
|  |  |  |  |  | 37960902 | cg08608193 |  |  |
|  |  |  |  |  | 37960974 | cg04403917 |  |  |
| FAM133A | Family With Sequence Similarity 133 Member A | It is a Protein Coding gene. | Cancer/Testis Antigen 115, Protein FAM133A, Family With Sequence Similarity 133, Member A, CT115. | X | 92928508 | cg21893284 | TSS1500;TSS1500;TSS1500;1stExon;TSS1500;5'UTR | chrX:92928546-92928786 |
|  |  |  |  |  | 92928608 | cg22189618 |  |  |
|  |  |  |  |  | 92928610 | cg19936436 |  |  |
| FAM155A | Family With Sequence Similarity 155 Member A | It is a Protein Coding gene associated with diseases such as; diverticulitis. | Transmembrane Protein FAM155A | 13 | 108520566 | cg24567424 | TSS1500 | chr13:108519292-108521063 |
|  |  |  |  |  | 108520827 | cg18588768 |  |  |
|  |  |  |  |  | 108520945 | cg22302985 |  |  |
| FLJ43390 | a.k.a. long intergenic non-protein coding RNA 643 | No published information found. | LINC00643; Putative UPF0730 Protein Encoded By LINC00643 | 14 | 62584037 | cg10227327 | TSS200 | chr14:62583679-62584279 |
|  |  |  |  |  | 62584278 | cg07471209 | Body |  |
|  |  |  |  |  | 62584513 | cg03479715 |  |  |
| FMN2 | Formin 2 | Critical regulator of p21/cell cycle progression. Hypermethylated in CRC. | Formin 2 | 1 | 240254960 | cg15748490 | TSS1500 | chr1:240254959-240257063 |
|  |  |  |  |  | 240254988 | cg19591056 | TSS200 |  |
|  |  |  |  |  | 240255136 | cg01535698 |  |  |
| FREM3 | FRAS1-related extracellular matrix protein 3 | Cell adhesion and molecular binding. Associated with Fraser syndrome 1. | FRAS1 Related Extracellular Matrix 3 | 4 | 144621385 | cg04514249 | 1stExon | chr4:144620822-144622218 |
|  |  |  |  |  | 144621898 | cg10056356 | TSS200 |  |
|  |  |  |  |  | 144621938 | cg08334984 |  |  |
|  |  |  |  |  | 144621945 | cg17495719 |  |  |
|  |  |  |  |  | 144621952 | cg09556043 |  |  |
|  |  |  |  |  | 144621971 | cg15042811 |  |  |
| GALR1 | Galanin Receptor 1 | Receptor for the hormone galanin. Its activity is mediated by G proteins which inhibit adenylate cyclase activity | GALNR1, GAL1-R, GALR-1, GALNR. | 18 | 74961966 | cg03659519 | TSS200 | chr18:74961556-74963822 |
|  |  |  |  |  | 74961968 | cg20872937 |  |  |
|  |  |  |  |  | 74962000 | cg17911318 |  |  |
|  |  |  |  |  | 74962133 | cg03502002 | 1stExon  5'UTR |  |
|  |  |  |  |  | 74962216 | cg01178451 |  |  |
|  |  |  |  |  | 74962369 | cg04534765 |  |  |
|  |  |  |  |  | 74962672 | cg06360427 | 1stExon |  |
|  |  |  |  |  | 74962794 | cg10390058 |  |  |
| GFRA1 | GDNF receptor alpha-1 | Receptor for GDNF. Mediates the GDNF-induced auto phosphorylation and activation of the RET receptor. | GDNF receptor alpha-1, GDNFR-alpha-1, GFR-alpha-1, RET ligand 1, TGF-beta-related neurotrophic factor receptor 1. | 10 | 118032879 | cg18672939 | 1stExon  5'UTR  TSS1500  TSS200 | chr10:118030732-118034230 |
|  |  |  |  |  | 118032892 | cg08194313 |  |  |
|  |  |  |  |  | 118032905 | cg27341926 |  |  |
| GRIA4 | Glutamate receptor 4 | Functions as ligand-gated ion channel in the central nervous system and plays an important role in excitatory synaptic transmission. Associated with Neurodevelopmental disorder with or without seizures and gait abnormalities. | GluR-4, GluR4, AMPA-selective glutamate receptor 4, GluR-D, Glutamate receptor ionotropic, AMPA 4, GluA4 | 11 | 105480979 | cg03243226 | 5'UTR  TSS1500  1stExon | chr11:105481126-105481422 |
|  |  |  |  |  | 105481283 | cg15603568 | 5'UTR;  TSS200 | chr11:105481126-105481422 |
|  |  |  |  |  | 105481292 | cg23559689 | 5'UTR;  TSS200 |  |
|  |  |  |  |  | 105481306 | cg00343633 |  |  |
|  |  |  |  |  | 105481317 | cg03225817 |  |  |
|  |  |  |  |  | 105481319 | cg04747226 |  |  |
|  |  |  |  |  | 105481322 | cg07972135 |  |  |
|  |  |  |  |  | 105481406 | cg21217024 |  |  |
|  |  |  |  |  | 105481509 | cg19343464 | 5'UTR  1stExon |  |
| KATNAL2 | Katanin p60 ATPase-containing subunit A-like 2 | KATNAL2 is a Protein Coding gene whose activity promote rapid reorganization of cellular microtubule arrays. Diseases associated with KATNAL2 include Tinea Capitis and Dermatophytosis. | Katanin Catalytic Subunit A Like 2, Katanin P60 Subunit A Like 2, Katanin P60 Subunit A-Like 2, P60 Katanin-Like 2. | 18 | 44526700 | cg12171183 | TSS200 | chr18:44526866-44527137 |
|  |  |  |  |  | 44526707 | cg06198398 |  |  |
|  |  |  |  |  | 44526743 | cg20469799 |  |  |
| KCNC2 | Potassium Voltage-Gated Channel Subfamily C Member 2 | Encodes for an integral membrane protein that mediates the voltage-dependent potassium ion permeability of excitable membranes primarily in the brain. Associated with spinocerebellar ataxia 13. | Potassium Voltage-Gated Channel, Shaw-Related Subfamily, Member 2, Voltage-Gated Potassium Channel Kv3.2, Shaw-Like Potassium Channel, KV3.2, | 12 | 75601711 | cg17203063 | Body | chr12:75601081-75601752 |
|  |  |  |  |  | 75601803 | cg25820699 | 5'UTR |  |
|  |  |  |  |  | 75601824 | cg10148473 |  |  |
| LOC645323 | Homo sapiens hypothetical LOC645323, mRNA | No published information found. | No published information found. | 5 | 87974369 | cg18318649 | Body;TSS200 | chr5:87976094-87976546 |
|  |  |  |  |  | 87974398 | cg13982529 | Body;TSS200 | chr5:87976094-87976546 |
|  |  |  |  |  | 87974414 | cg21230745 | Body  TSS200 | chr5:87976094-87976546 |
| MARCH11 | Membrane associated ring-CH-type finger 11 | Modulates lysosomal degradation and delivery. | E3 Ubiquitin-Protein Ligase MARCH11, MARCH-XI, Membrane Associated Ring Finger 11, RNF226. | 5 | 16180068 | cg17712694 | TSS200 | chr5:16179064-16180420 |
|  |  |  |  |  | 16180072 | cg16150752 |  |  |
|  |  |  |  |  | 16180076 | cg21901718 |  |  |
|  |  |  |  |  | 16180259 | cg18325622 | TSS1500 |  |
|  |  |  |  |  | 16180266 | cg23065934 |  |  |
| MIR129-2 | MicroRNA 129-2 | MIR129-2 is a tumor suppressive miRNA methylated in lymphoid malignancies. Diseases associated with MIR129-2 include Retinoblastoma and Univentricular Heart. Found also to be hypermethylated in colorectal cancer cell lines. | Hsa-Mir-129-2, Mir-129-2, MIRN129-2, MIR-129b. | 11 | 43602845 | cg15556502 | TSS200 | chr11:43602545-43603215 |
|  |  |  |  |  | 43602847 | cg14416371 |  |  |
|  |  |  |  |  | 43602857 | cg14944647 |  |  |
|  |  |  |  |  | 43602879 | cg01939477 |  |  |
|  |  |  |  |  | 43602914 | cg16407471 |  |  |
|  |  |  |  |  | 43602920 | cg05376374 |  |  |
|  |  |  |  |  | 43602965 | cg03365311 | Body |  |
| MIR663 | MicroRNA 663a | MicroRNA 663a is a short non-coding RNA that are involved in post-transcriptional regulation of gene expression in multicellular organisms by affecting both the stability and translation of mRNAs. Diseases associated with MIR663A include Systemic Lupus Erythematosus, breast cancer and Ovarian Cancer. Hypermethylated in endometrial cancer. | MicroRNA 663, Hsa-Mir-663a, Hsa-Mir-663, Mir-663a, MIRN663, MIR663. | 20 | 26188971 | cg06007966 | TSS200 | chr20:26188638-26190348 |
|  |  |  |  |  | 26188976 | cg01521987 |  |  |
|  |  |  |  |  | 26188997 | cg08304190 |  |  |
|  |  |  |  |  | 26190328 | cg04150495 | TSS1500 |  |
|  |  |  |  |  | 26190347 | cg20395967 |  |  |
|  |  |  |  |  | 26190354 | cg10715092 |  |  |
| MSC | Musculin | MSC is a protein coding gene that encodes for a protein known as a lineage-restricted transcriptional repressor in muscle differentiation. May play a role in regulating antigen-dependent B-cell differentiation. No disorders were found for MSC Gene. | Class A Basic Helix-Loop-Helix Protein 22, Activated B-Cell Factor-1, BHLHa22, ABF-1, ABF1, Activated B-Cell Factor 1, Homolog Of Mouse Musculin, Musculin (Activated B-Cell Factor-1), Activated B-Cell Factor 1, BHLHA22, MYOR. | 8 | 72754953 | cg26799209 | Body | chr8:72755783-72756667 |
|  |  |  |  |  | 72755052 | cg13620034 |  |  |
|  |  |  |  |  | 72755162 | cg26876974 |  |  |
|  |  |  |  |  | 72756058 | cg25832771 | 1stExon |  |
|  |  |  |  |  | 72756155 | cg09734791 |  |  |
|  |  |  |  |  | 72756341 | cg14409559 |  |  |
| NELL1 | Neural EGFL Like 1 | This gene encodes a cytoplasmic protein which may be involved in cell growth regulation and differentiation. Associated diseases are: fibrosarcomatous osteosarcoma, craniosynostosis, gnathodiaphyseal dysplasia and synostosis. Hypermethylated in esophageal adenocarcinoma. | Protein Kinase C-Binding Protein NELL1, Nel-Related Protein 1, NRP1, Neural Epidermal Growth Factor-Like 1, Nel (Chicken)-Like 1, NEL-Like 1 (Chicken), NEL-Like Protein 1, IDH3GL. | 11 | 20690628 | cg22769941 | TSS1500 | chr11:20690579-20691845 |
|  |  |  |  |  | 20690682 | cg06873316 |  |  |
|  |  |  |  |  | 20690720 | cg14749465 |  |  |
|  |  |  |  |  | 20690807 | cg02510267 |  |  |
|  |  |  |  |  | 20690930 | cg12071328 | TSS200 |  |
| NID2 | Nidogen 2 | Cell-adhesion protein involved in maintaining basement membrane structure. NID2 is methylated in human gastrointestinal cancer and also serves as an early detection methylated biomarker for oral SCC and bladder cancer. | Osteonidogen, Nidogen-2, NID-2 | 14 | 52535178 | cg14897833 | Body | chr14:52534581-52536722 |
|  |  |  |  |  | 52535425 | cg07975778 |  |  |
|  |  |  |  |  | 52535758 | cg13592399 | 5'UTR  1stExon |  |
|  |  |  |  |  | 52536066 | cg26394244 | TSS200 |  |
|  |  |  |  |  | 52536147 | cg22881914 | TSS1500 |  |
|  |  |  |  |  | 52536175 | cg04701034 |  |  |
| NKX2-6 | NK2 Homeobox 6 | Essential for development of the heart-like dorsal vessel. Diseases associated with NKX2-6 include Conotruncal Heart Malformations and Familial Atrial Fibrillation. Methylated in CRC. | NKX2F, NK2 Transcription Factor Related, Locus 6, Homeobox Protein NK-2 Homolog F, Homeobox Protein NK2 Homolog F, Tinman Paralog (Drosophila), Homeobox Protein NKX2.6, Tinman Paralog, NKX4-2, CSX2, CTHM. | 8 | 23563859 | cg13021619 | 1stExon | chr8:23562475-23565175 |
|  |  |  |  |  | 23563925 | cg14428146 | TSS200 |  |
|  |  |  |  |  | 23563970 | cg15854847 |  |  |
| NOVA1 | NOVA Alternative Splicing Regulator 1 | NOVA1 is a Protein Coding gene involved in nucleic acid binding and RNA binding. Diseases associated with NOVA1 include Partial Fetal Alcohol Syndrome. Differentially methylated in nasal polyps. | Neuro-Oncological Ventral Antigen 1, Ventral Neuron-Specific Protein 1, Onconeural Ventral Antigen 1, RNA-Binding Protein Nova-1, Paraneoplastic Ri Antigen, Nova-1. | 14 | 27066634 | cg18488855 | 1stExon | chr14:27066313-27066578 |
|  |  |  |  |  | 27066771 | cg15602241 | 5'UTR  1stExon |  |
|  |  |  |  |  | 27067372 | cg20478129 | TSS1500 |  |
|  |  |  |  |  | 27067895 | cg07543626 |  |  |
|  |  |  |  |  | 27068093 | cg16791424 |  |  |
|  |  |  |  |  | 27068135 | cg23502778 |  |  |
| NPTX2 | Neuronal Pentraxin 2 | Protein coding gene involved in excitatory synapse formation. Diseases associated with NPTX2 include Narcolepsy and Kearns-Sayre Syndrome. | Neuronal Pentraxin II, Neuronal Pentraxin-2, Apexin, NP-II, NP2, Neuronal Activity-Regulated Pentaxin, NARP. | 7 | 98246001 | cg13314145 | TSS1500 | chr7:98245805-98247759 |
|  |  |  |  |  | 98246006 | cg08315202 |  |  |
|  |  |  |  |  | 98246633 | cg18952796 | 5'UTR;1stExon |  |
| NPY | Neuropeptide Y | Protein Coding gene that influences many physiological processes, including cortical excitability, stress response, food intake, circadian rhythms, and cardiovascular function. Associated diseases include breath-holding spells and autonomic neuropathy. Hypermethylated in CRC. | Prepro-Neuropeptide Y, Pro-Neuropeptide Y, PYY4. | 7 | 24323799 | cg16964348 | TSS200 | chr7:24323558-24325080 |
|  |  |  |  |  | 24323840 | cg25884711 | 5'UTR  1stExon |  |
|  |  |  |  |  | 24323939 | cg21097881 | 5'UTR |  |
| NPY5R | Neuropeptide Y receptor type 5 | The activity of this receptor is mediated by G proteins that inhibit adenylate cyclase activity. Seems to be associated with food intake, food disorders, lipid metabolism and obesity. NPY5R is methylated in head and neck cancer. | Neuropeptide Y Receptor Type 5, NPY-Y5 Receptor, Y5 Receptor, NPYY5-R, NPY5-R, NPYR5. | 4 | 164265004 | cg20618622 | TSS200 | chr4:164264821-164265772 |
|  |  |  |  |  | 164265012 | cg08346159 |  |  |
|  |  |  |  |  | 164265442 | cg10341154 | 5'UTR |  |
| OLIG2 | Oligodendrocyte transcription factor 2 | Required for oligodendrocyte differentiation and development of somatic motor neurons in the hindbrain. Associated diseases are: oligodendroglioma, T-cell acute lymphoblastic leukemia, and brain cancer. | Protein Kinase C-Binding Protein 2, PRKCBP2, BHLHe19, BHLHB1, RACK17, Protein Kinase C-Binding Protein RACK17, Protein Kinase C Binding Protein 2, BHLHE19, OLIGO2, Oligo2, BHLHb1. | 21 | 34398199 | cg08870743 | TSS200 | chr21:34395128-34400245 |
|  |  |  |  |  | 34398222 | cg23253569 |  |  |
|  |  |  |  |  | 34398226 | cg27357571 |  |  |
| ONECUT2 | One Cut Homeobox 2 | Acts as a transcriptional activator particularly in neuroendocrine tumors. Associated with suppurative thyroiditis disorder. | Hepatocyte Nuclear Factor 6-Beta, One Cut Domain Family Member 2, Transcription Factor ONECUT-2, HNF-6-Beta, OC-2, ONECUT-2 Homeodomain Transcription Factor, One Cut Domain, Family Member 2, Onecut 2, HNF6B, OC2. | 18 | 55108537 | cg20956738 | Body | chr18:55103154-55108853 |
|  |  |  |  |  | 55108852 | cg10835584 |  |  |
|  |  |  |  |  | 55108947 | cg16941302 |  |  |
| OTX2OS1 | OTX2 Antisense RNA 1 (Head To Head) | Methylated in lung cancer cell lines and also in some healthy lung tissues. | OTX2 Antisense RNA 1 (Non-Protein Coding), Otx2 Opposite Strand Transcript 1, OTX2 Antisense RNA 1. | 14 | 57278710 | cg09624466 | TSS1500 | chr14:57278709-57279116 |
|  |  |  |  |  | 57278729 | cg21039708 |  |  |
|  |  |  |  |  | 57279115 | cg25092838 |  |  |
|  |  |  |  |  | 57279275 | cg23478293 |  |  |
| PAX1 | Paired box 1 | Frequent methylation in HNSCC. Shown to segregate normal and neoplastic cervical cancer. Gene is silenced by methylation in ovarian and cervical cancers and may be a tumor suppressor gene | Paired Box Gene 1, HUP48, Paired Domain Gene HuP48, HuP48, OFC2. | 20 | 21686273 | cg19054524 | TSS200 | chr20:21686199-21687689 |
|  |  |  |  |  | 21686282 | cg08448701 |  |  |
|  |  |  |  |  | 21686293 | cg01783070 |  |  |
|  |  |  |  |  | 21686308 | cg19079845 | 1stExon  5'UTR |  |
| PAX6 |  | Responsible for formation of organs, tissues and neuron development during embryonic development. Also maintains certain cells after birth such as the olfactory bulb and eyes. Associated diseases include: Aniridia, Peters anomaly and WAGR syndrome. | AN, AN2, D11S812E, MGC17209, MGDA, paired box gene 6, paired box gene 6 isoform a, paired box gene 6 isoform b, PAX6_HUMAN | 11 | 31825756 | cg11827910 | Body | chr11:31825743-31826967 |
|  |  |  |  | 11 | 31825792 | cg20014398 |  |  |
|  |  |  |  |  | 31825833 | cg11128216 |  |  |
|  |  |  |  |  | 31825969 | cg22982368 |  |  |
|  |  |  |  |  | 31826421 | cg11469061 |  |  |
|  |  |  |  |  | 31826574 | cg09537620 |  |  |
|  |  |  |  |  | 31827084 | cg18372607 |  |  |
| PCDH10 | Protocadherin | Potential calcium-dependent cell-adhesion protein. Associated with autism and epileptic encephalopathy. PCDH10 methylation is also associated with gastric cancer, nasopharyngeal carcinoma and esophageal carcinoma. | Protocadherin-10, KIAA1400, OL-PCDH, PCDH19. | 4 | 134070369 | cg02562431 | TSS200 | chr4:134069162-134070442 |
|  |  |  |  |  | 134070389 | cg12746059 |  |  |
|  |  |  |  |  | 134070416 | cg27600205 |  |  |
| PDE1C | Phosphodiesterase 1C | Regulates smooth muscle cell proliferation and the stability of growth factor receptors. Associated with deafness, autosomal dominant 74. | Calcium/Calmodulin-Dependent 3',5'-Cyclic Nucleotide Phosphodiesterase 1C 3 4 Phosphodiesterase 1C, Calmodulin-Dependent 70kDa 2 3 3',5'-Cyclic-AMP Phosphodiesterase 3 4 3',5'-Cyclic-GMP Phosphodiesterase 3 4 Cam-PDE 1C 3 4 Hcam3 3 4 Human 3',5' Cyclic Nucleotide Phosphodiesterase (HSPDE1C1A) 3 Phosphodiesterase 1C, Calmodulin-Dependent (70kD) DFNA74 3 HCam-3 3 | 7 | 32110650 | cg15988350 | 5'UTR | chr7:32110063-32110910 |
|  |  |  |  |  | 32110988 | cg22131691 | 1stExon  5'UTR |  |
|  |  |  |  |  | 32111001 | cg10989138 | TSS200 |  |
| PENK | Proenkephalin | They play a role in a number of physiologic functions, including pain perception and responses to stress. Associated with drug dependence disorders and bone cancer. | Preproenkephalin, Proenkephalin-A, Enkephalin A, Peptide F, PENK-A, PE. | 8 | 57358625 | cg18742346 | 5'UTR  TSS200 | chr8:57358126-57359415 |
|  |  |  |  |  | 57358651 | cg03483150 |  |  |
|  |  |  |  |  | 57358713 | cg04612444 |  |  |
| PEX5L | Peroxisomal biogenesis factor 5 like | Trafficking of peroxisomal matrix proteins | TRIP8b, PEX5R, PXR2, Pex5p-Related Protein, PEX5RP, Pex5Rp, PXR2B. | 3 | 179755086 | cg18780412 | TSS1500 | chr3:179754520-179755245 |
|  |  |  |  |  | 179755235 | cg21176048 |  |  |
|  |  |  |  |  | 179755285 | cg05131623 |  |  |
| RALYL | RALY RNA Binding Protein Like | Protein binding gene widely expressed in brain. | Heterogeneous Nuclear Ribonucleoprotein C-Like 3, RNA-Binding Raly-Like Protein, HnRNP Core Protein C-Like 3, HNRPCL3, HRALYL. | 8 | 85094742 | cg09757712 | TSS1500 | chr8:85096759-85097247 |
|  |  |  |  |  | 85094842 | cg08327106 |  |  |
|  |  |  |  |  | 85094904 | cg01134282 |  |  |
| RFX4 | Regulatory Factor X4 | Transcription factor that plays a role in early brain development. No disorders were found for RFX4 Gene. | Regulatory Factor X, 4, Testis Development Protein NYD-SP10, Transcription Factor RFX4, Winged-Helix Transcription Factor RFX4, Regulatory Factor X 4, NYD-SP10. | 12 | 106979430 | cg21961487 | Body | chr12:106979429-106981086 |
|  |  |  |  |  | 106979451 | cg00379648 |  |  |
|  |  |  |  |  | 106979482 | cg10040329 |  |  |
| RYR2 | Ryanodine receptor 2 | Mediates cellular calcium release | Islet-Type Ryanodine Receptor, HRYR-2, ARVC2, VTSIP, ARVD2, RyR2, RyR. | 1 | 237205409 | cg18375860 | TSS1500 | chr1:237205126-237206644 |
|  |  |  |  |  | 237205950 | cg11657808 | Body |  |
|  |  |  |  |  | 237205999 | cg07914084 |  |  |
| SALL1 | Spalt Like Transcription Factor 1 | Transcriptional repressor involved in organogenesis. Associated with townes-brocks syndrome and hemifacial microsomia. | Zinc Finger Protein Spalt-1, Zinc Finger Protein SALL1, Zinc Finger Protein 794, Sal-Like Protein 1, ZNF794, Sal-1, Epididymis Secretory Protein Li 89, Spalt-Like Transcription Factor 1, Sal (Drosophila)-Like 1, Sal-Like 1 (Drosophila), HEL-S-89, HSAL1, HSal1, SAL1, TBS. | 16 | 51184355 | cg04550052 | Body  5'UTR  1stExon | chr16:51183699-51188763 |
|  |  |  |  |  | 51184379 | cg04698114 |  |  |
|  |  |  |  |  | 51184392 | cg00582524 |  |  |
|  |  |  |  |  | 51184562 | cg08526074 | Body  TSS200 |  |
| SDF4 | Stromal Cell Derived Factor 4 | SDF4 is a protein coding gene involved in regulating calcium dependent cellular activities. Diseases associated with SDF4 include Ehlers-Danlos Syndrome Progeroid Type and Shrimp Allergy. | 45 KDa Calcium-Binding Protein, Cab45, SDF-4, Stromal Cell-Derived Factor 4, Calcium Binding Protein, CAB45. | 1 | 1168432 | cg13924635 | TSS1500  1^st^ Exon | chr1:1167001-1168985 |
|  |  |  |  |  | 1168541 | cg22220310 |  |  |
|  |  |  |  |  | 1168550 | cg14477263 |  |  |
| SIAH3 | Siah E3 Ubiquitin Protein Ligase Family Member 3 | E3 ubiquitin-protein ligase that mediates ubiquitination and subsequent proteasomal degradation of target proteins. | Seven In Absentia Homolog 3, Siah-3. | 13 | 46425837 | cg02674384 | 5'UTR  1^st^ Exon |  |
|  |  |  |  |  | 46425866 | cg08018585 | TSS200 |  |
|  |  |  |  |  | 46425874 | cg26667946 |  |  |
| SIM1 | SIM BHLH Transcription Factor 1 | Transcriptional factor that may have pleiotropic effects during embryogenesis and in the adult. Associated with obesity and down syndrome. | Single-Minded Family BHLH Transcription Factor 1, Class E Basic Helix-Loop-Helix Protein 14, Single-Minded Homolog 1, BHLHe14, BHLHE14. | 6 | 100911687 | cg11471772 | TSS200 | chr6:100912071-100913337 |
|  |  |  |  |  | 100911701 | cg04927931 |  |  |
|  |  |  |  |  | 100911709 | cg21063722 |  |  |
| SIX6 | SIX Homeobox 6 | May be involved in eye development. Associated with optic disc anomalies with retinal and/or macular dystrophy. | Optic Homeobox 2, OPTX2, Sine Oculis Homeobox-Like Protein 6, Sine Oculis Homeobox Protein 6, MCOPCT2, ODRMD, SIX9 | 14 | 60975846 | cg18639233 | TSS200 | chr14:60975732-60978180 |
|  |  |  |  |  | 60975912 | cg20585530 |  |  |
|  |  |  |  |  | 60975964 | cg06785999 | 1stExon  5'UTR |  |
| SLC46A3 | Solute carrier family 46 member 3 | Transmembrane protein family responsible for transporting small molecules across membranes | Solute Carrier Family 46, Member 3, FKSG16. | 13 | 29293213 | cg20752818 | TSS200 | chr13:29292670-29293329 |
|  |  |  |  |  | 29293245 | cg21789898 |  |  |
|  |  |  |  |  | 29293279 | cg18682423 |  |  |
|  |  |  |  |  | 29293325 | cg02233559 |  |  |
| SLITRK1 | SLIT And NTRK Like Family Member 1 | It is involved in synaptogenesis and promotes excitatory synapse differentiation. Associated with gilles de la tourette syndrome and obsessive-compulsive disorder. | Leucine-Rich Repeat-Containing Protein 12, LRRC12, SLIT And NTRK-Like Family, Member 1, Slit And Trk Like Gene 1, KIAA1910, TTM. | 13 | 84456127 | cg07104706 | 1stExon  5'UTR | chr13:84453664-84453897 |
|  |  |  |  |  | 84456171 | cg19696317 |  |  |
|  |  |  |  |  | 84456258 | cg26998274 |  |  |
|  |  |  |  |  | 84456308 | cg20312205 |  |  |
| SORCS3 | Sortilin related VPS10 domain containing receptor 3 | Frequently methylated in gastric cancer | VPS10 Domain Receptor Protein SORCS 3, KIAA1059, SORCS | 10 | 106400880 | cg16787600 | 1stExon  5'UTR | chr10:106399567-106402812 |
|  |  |  |  |  | 106401319 | cg10778841 | 1stExon |  |
|  |  |  |  |  | 106401479 | cg18326021 |  |  |
| ST8SIA5 | ST8 Alpha-N-Acetyl-Neuraminide Alpha-2,8-Sialyltransferase 5 | May be involved in the synthesis of gangliosides. Associated with spastic paraplegia disorder. | SIAT8E, Alpha-2, 8-Sialyltransferase 8E, Sialyltransferase St8Sia V, SIAT8-E, ST8SiaV. | 18 | 44337853 | cg26155939 | TSS1500 | chr18:44337510-44338100 |
|  |  |  |  |  | 44337910 | cg15049968 |  |  |
|  |  |  |  |  | 44337922 | cg23129478 |  |  |
| T | T-Box Transcription Factor T | Involved in the transcriptional regulation of genes required for mesoderm formation and differentiation. Associated with spinal bifida. | T Brachyury Transcription Factor, Brachyury Protein, T, Brachyury Homolog, SAVA, TFT. | 6 | 166582206 | cg19675288 | TSS200 | chr6:166579973-166583423 |
|  |  |  |  |  | 166582310 | cg06073449 |  |  |
|  |  |  |  |  | 166582393 | cg06463958 | TSS1500 |  |
| TAC1 | Tachykinin Precursor 1 | Tachykinins excite neurons, evoke behavioral responses, are potent vasodilators and secretagogues, and contract many smooth muscles. Associated with bronchitis. | Substance P, NKNA, TAC2, PPT, Neurokinin A, Tachykinin 2, Hs.2563, NK2, NPK, NKA, Protachykinin-1. | 7 | 97361241 | cg01287975 | TSS200 | chr7:97361132-97363018 |
|  |  |  |  |  | 97361244 | cg11873482 |  |  |
|  |  |  |  |  | 97361252 | cg17437939 |  |  |
| TCP11 | T-complex protein 11 homolog | Plays a role in the process of sperm capacitation and acrosome reactions. | T-Complex 11, T-Complex 11- Testis-Specific, Testis Secretory Sperm-Binding Protein Li 222n, T-Complex 11 Homolog, D6S230E, FPPR. | 6 | 35109398 | cg24623244 | TSS1500 | chr6:35108801-35109499 |
|  |  |  |  |  | 35109435 | cg12090052 |  |  |
|  |  |  |  |  | 35109485 | cg12835524 |  |  |
| TMEM132C | Transmembrane protein 132C | TMEM132C is a Protein Coding gene. Associated with adolescent idiopathic scoliosis and Alzheimer’s disease. | Protein Phosphatase 1, Regulatory Subunit 152, PPP1R152 | 12 | 128752040 | cg04475027 | Body | chr12:128751041-128753151 |
|  |  |  |  |  | 128752058 | cg26682580 |  |  |
|  |  |  |  |  | 128752246 | cg12868067 |  |  |
|  |  |  |  |  | 128752356 | cg11877129 |  |  |
| TMEM155 | Transmembrane Protein 155 | Protein Coding gene associated with type II diabetes mellitus. | Protein TMEM155. | 4 | 122686432 | cg03227184 | TSS200  Body | chr4:122685860-122686565 |
|  |  |  |  |  | 122686453 | cg04638468 |  |  |
|  |  |  |  |  | 122686456 | cg08553437 |  |  |
|  |  |  |  |  | 122686493 | cg07978472 |  |  |
| TRAM1L1 | Translocation Associated Membrane Protein 1 Like 1 | Required for the translocation of secretory proteins across the ER membrane. | Translocating Chain-Associated Membrane Protein 1-Like 1. | 4 | 118006812 | cg11163975 | TSS200 | chr4:118006538-118006859 |
|  |  |  |  |  | 118006825 | cg10277927 |  |  |
|  |  |  |  |  | 118006832 | cg01904183 |  |  |
| TRH | Thyrotropin Releasing Hormone | It controls the secretion and regulation of thyroid-stimulating hormone. Associated with hypothyroidism. | Prothyroliberin, TRF, Protirelin, TSH-releasing factor, Thyroliberin, Thyrotropin-releasing factor | 3 | 129693370 | cg18862481 | 5'UTR  1stExon | chr3:129693127-129694841 |
|  |  |  |  |  | 129693385 | cg11940285 |  |  |
|  |  |  |  |  | 129693489 | cg22512438 |  |  |
|  |  |  |  |  | 129693586 | cg02700891 |  |  |
|  |  |  |  |  | 129693613 | cg01009664 |  |  |
| WDR17 | WD repeat domain 17 | Frequently altered in T cell malignancies | WD Repeat-Containing Protein 17, Epididymis Secretory Sperm Binding Protein. | 4 | 176987020 | cg08095852 | 1stExon  5'UTR | chr4:176986921-176987360 |
|  |  |  |  |  | 176987174 | cg27486637 |  |  |
|  |  |  |  |  | 176987313 | cg08684639 | 5'UTR |  |
| ZIC4 | Zic Family Member 4 | ZIC4 binds to DNA. Diseases associated with ZIC4 include Isolated Dandy-Walker Malformation With Hydrocephalus. | Zinc Finger Protein Of The Cerebellum 4, Zinc Finger Protein ZIC 4, Zinc Family Member 4 Protein HZIC4. | 3 | 147106010 | cg00334063 | 3'UTR  Body | chr3:147108511-147111703 |
|  |  |  |  |  | 147106208 | cg22614239 |  |  |
|  |  |  |  |  | 147106489 | cg08889797 |  |  |
|  |  |  |  |  | 147111660 | cg03900143 | Body  TSS1500 |  |
|  |  |  |  |  | 147112081 | cg02653559 | Body |  |
|  |  |  |  |  | 147112096 | cg26014036 |  |  |
| ZIK1 | Zinc finger protein interacting with K protein 1 | Methylated biomarker for esophageal SCC. | Zinc Finger Protein Interacting With Ribonucleoprotein K, Zinc Finger Protein 762, ZNF762. | 19 | 58095581 | cg12060744 | TSS200 | chr19:58094739-58095764 |
|  |  |  |  |  | 58095588 | cg01046104 |  |  |
|  |  |  |  |  | 58095595 | cg18579862 |  |  |
|  |  |  |  |  | 58095659 | cg26246807 | 5'UTR  1stExon |  |
| ZNF154 | zinc finger protein 154 | Hypermethylation in 15 of 16 distinct cancer types from TCGA | Zinc Finger Protein 154 (PHZ-92), KIAA2003, PHZ-92. | 19 | 58220370 | cg05661282 | 5'UTR  1stExon | chr19:58220189-58220517 |
|  |  |  |  |  | 58220494 | cg21790626 |  |  |
|  |  |  |  |  | 58220516 | cg27049766 |  |  |
|  |  |  |  |  | 58220657 | cg03234186 | TSS200 |  |
|  |  |  |  |  | 58220662 | cg08668790 |  |  |
|  |  |  |  |  | 58220718 | cg12506930 |  |  |
| ZNF177 | Zinc finger protein 177 | Methylated in gastric and hepatocellular cancer | PIGX | 19 | 9473598 | cg05928342 | TSS200 | chr19:9473589-9474001 |
|  |  |  |  |  | 9473674 | cg13703871 |  |  |
|  |  |  |  |  | 9473684 | cg08065231 |  |  |
| ZNF529 | Zinc finger protein 529 | No published information on this gene | KIAA1615 | 19 | 37096321 | cg02587316 | TSS200  5'UTR | chr19:37095680-37096589 |
|  |  |  |  |  | 37096323 | cg18630667 |  |  |
|  |  |  |  |  | 37096329 | cg05020604 |  |  |
| ZNF542 | Zinc Finger Protein 542 | May be involved in transcriptional regulation. | Zinc Finger Protein 542 Pseudogene, Putative Zinc Finger Protein 542, Zinc Finger Protein Pseudogene | 19 | 56879645 | cg27477373 | TSS200  Body | chr19:56879417-56879995 |
|  |  |  |  |  | 56879662 | cg03146949 |  |  |
|  |  |  |  |  | 56879933 | cg08697092 | Body |  |
|  |  |  |  |  |  |  |  |  |
| ZNF582 | Zinc Finger Protein 582 | May be involved in transcriptional regulation. Associated with anal and cervical cancer. | N/A | 19 | 56905032 | cg20984085 | TSS200 | chr19:56904636-56905355 |
|  |  |  |  |  | 56905094 | cg25267765 | TSS1500 |  |
|  |  |  |  |  | 56905152 | cg07135042 |  |  |
| ZNF781 | Zinc Finger Protein 781 | Protein Coding gene that may be involved in transcriptional regulation. | N/A | 19 | 38183253 | cg03611452 | TSS200 | chr19:38182793-38183327 |
|  |  |  |  |  | 38183259 | cg25324105 |  |  |
|  |  |  |  |  | 38183262 | cg14587524 |  |  |
| ZNF788 | Zinc Finger Family Member 788. | Nucleic acid binding functions. Associated with coronary artery disease. | Zinc Finger Family Member 788, Pseudogene, Putative KRAB Domain-Containing Protein ZNF788. | 19 | 12203198 | cg06384763 | Body | chr19:12203028-12203503 |
|  |  |  |  |  | 12203315 | cg01172903 |  |  |
|  |  |  |  |  | 12203349 | cg11767984 |  |  |
| ZSCAN1 | Zinc Finger And SCAN Domain Containing 1 | Associated with Desbuquois dysplasia: short stature, joint laxity, developmental delay, hand anomalies, fusion protein with EV4 in prostate cancer. Hypermethylated in Cervical Cancer. | SCAN1, CANT1 | 19 | 58545122 | cg11312896 | TSS1500 | chr19:58545115-58545897 |
|  |  |  |  |  | 58545149 | cg21331821 |  |  |
|  |  |  |  |  | 58545160 | cg24368848 |  |  |
| ZSCAN18 | Zinc Finger And SCAN Domain Containing 18 | Functions of this gene include nucleic acid binding and DNA-binding transcription factor activity. Frequently methylated in gastrointestinal cancer. | Zinc Finger Protein 447, Zinc Finger And SCAN Domain-Containing Protein 18, ZNF447. | 19 | 58609730 | cg22031998 | Body  TSS200 | chr19:58609338-58609988 |
|  |  |  |  |  | 58609744 | cg18428688 |  |  |
|  |  |  |  |  | 58609764 | cg07657064 |  |  |
|  |  |  |  |  | 58609770 | cg23229395 |  |  |
