# Supplementary material for: Genome-wide host methylation profiling of anal and cervical carcinoma
Source: PLoS One. 2021 Dec 9;16(12):e0260857. doi: 10.1371/journal.pone.0260857 (PMC8659695; doi:10.1371/journal.pone.0260857)
Supplement: S3 Table — By DMR-defining criteria, 36 separate genes comprised of 173 CpG loci that significantly distinguished CC from normal cervical tissue were identified. (DOCX) [file pone.0260857.s005.docx]

**S3 Table. Cervical Cancer Genes**

| **Gene Symbol** | **Product** | **Function /Association with Disease** | **Aliases** | **Chr.** | **Position** | **CpG Number** | **Genomic Region** | **Island** |
| --- | --- | --- | --- | --- | --- | --- | --- | --- |
|  |  |  |  |  |  |  |  |  |
| ASCL1 | Achaete-scute family bHLH transcription factor 1 | Among a panel of methylated genes for the detection of oral SCC. Methylated in anal cancer. | HASH1; Class A Basic Helix-Loop-Helix Protein 46, Achaete-Scute Homolog 1, BHLHa46, ASH-1, BHLHA46, MASH1 | 12 | 103352235 | cg02246645 | 1stExon | chr12:103351579-103352695 |
|  |  |  |  |  | 103352267 | cg27420520 |  |  |
|  |  |  |  |  | 103352294 | cg20718350 |  |  |
|  |  |  |  |  | 103352326 | cg03700449 |  |  |
|  |  |  |  |  | 103352454 | cg22356339 |  |  |
| ATP10A | ATPase phospholipid transporting 10A (putative) | Possible imprinted gene. Methylated in colorectal cancer. | ATP10C, ATPVA, ATPVC, EC 3.6.3.1, EC 7.6.2.1, KIAA0566, EC 3.6.3, ATPase Type IV, Phospholipid Transporting (P-Type), Phospholipid-Transporting ATPase VA. | 15 | 26108263 | cg20174066 | 1stExon.  5'UTR | chr15:26107503-26108818 |
|  |  |  |  |  | 26108391 | cg03419058 | TSS200 |  |
|  |  |  |  |  | 26108399 | cg16389285 |  |  |
|  |  |  |  |  | 26108401 | cg20124450 |  |  |
|  |  |  |  |  | 26108410 | cg22113930 |  |  |
|  |  |  |  |  | 26108412 | cg26230285 |  |  |
| BARHL2 | BarH like homeobox 2 | DNA-binding transcription activator activity, RNA polymerase II-specific. Hypermethylated in astrocytomas brain tumor but not in normal brain tissue. | BarH-Like 2 Homeobox Protein 3 4 BarH (Drosophila)-Like 2, BarH-Like Homeobox 2. | 1 | 91183051 | cg25026529 | TSS1500 | chr1:91183240-91184540 |
|  |  |  |  |  | 91183519 | cg13939859 |  |  |
|  |  |  |  |  | 91183697 | cg11823511 |  |  |
|  |  |  |  |  | 91184126 | cg20311863 |  |  |
| CCDC81 | coiled-coil domain containing 81 | Protein coding. Associated with a digestive system disease known as Eosinophilic esophagitis. Hypermethylated in endometrial tumors. | FLJ16339, FLJ23514 | 11 | 86085694 | cg18282849 | TSS200 | chr11:86085693-86086006 |
|  |  |  |  |  | 86085715 | cg10395685 |  |  |
|  |  |  |  |  | 86085757 | cg18121003 |  |  |
|  |  |  |  |  | 86085932 | cg23817893 | 1stExon  5'UTR |  |
|  |  |  |  |  | 86086005 | cg04573398 |  |  |
| CLIC6 | chloride intracellular channel 6 | Associated with gastric diseases. Methylation identified in cervical cancer but not verified. | Chloride Intracellular Channel Protein 6, Parchorin, CLIC1L, Chloride Channel Form A | 21 | 36041605 | cg19200589 | TSS200 | chr21:36041305-36043224 |
|  |  |  |  |  | 36041612 | cg18074297 |  |  |
|  |  |  |  |  | 36041683 | cg11528328 |  |  |
|  |  |  |  |  | 36041699 | cg10722799 | 1stExon |  |
| DPP10 | Dipeptidyl Peptidase Like 10 | Methylated in nicotine-exposed fetal lung and placental tissue. May be associated with colorectal cancer. Seen in Autism, mood disorder or schizophrenia. | DPRP-3, DPP X, DPRP3, DPL2, KIAA1492, DPPY, Dipeptidyl-Peptidase 10. | 2 | 115919785 | cg04075191 | Body;1stExon;5'UTR | chr2:115918737-115920765 |
|  |  |  |  |  | 115919829 | cg01718116 |  |  |
|  |  |  |  |  | 115919950 | cg00089091 | Body |  |
|  |  |  |  |  | 115920221 | cg13777681 |  |  |
| FMN2 | Formin 2 | Critical regulator of p21/cell cycle progression. Hypermethylated in CRC. | Formin-2 | 1 | 240254988 | cg19591056 | TSS200 | chr1:240254959-240257063 |
|  |  |  |  |  | 240255136 | cg01535698 |  | chr1:240254959-240257063 |
|  |  |  |  |  | 240255377 | cg02574509 | 5'UTR;1stExon | chr1:240254959-240257063 |
|  |  |  |  |  | 240255486 | cg25208017 | 1stExon | chr1:240254959-240257063 |
| GRK7 | G Protein-Coupled Receptor Kinase | Encodes a member of the guanine nucleotide-binding protein (G protein)-coupled receptor kinase subfamily of the Ser/Thr protein kinase family and is specifically expressed in the retina. Secretes rhodopsin kinase which controls shutoff of the photo response and adaptation to changing light conditions. | G Protein-Coupled Receptor Kinase, Rhodopsin Kinase, GPRK7, EC 2.7.11.16, EC 2.7.11.14, EC 2.7.11 | 3 | 141516232 | cg04472725 | Body | chr3:141516055-141516639 |
|  |  |  |  |  | 141516271 | cg18768784 |  |  |
|  |  |  |  |  | 141516291 | cg06822689 |  |  |
|  |  |  |  |  | 141516458 | cg12668399 |  |  |
| HLA-E | Major Histocompatibility Complex, Class I, E | Associated with mental disorders such as; Schizophrenia, Bipolar disorder and skin disorders such as; Parapsoriasis, Erythroderma, Maculopapular | Major Histocompatibility Complex, Class I, E, HLA Class I Histocompatibility Antigen, Alpha Chain E, MHC Class I Antigen E, HLA-6.2, MHC Class Ib Antigen, HLAE, QA1 | 6 | 30458519 | cg13007871 | Body | chr6:30457369-30458175 |
|  |  |  |  |  | 30458586 | cg00340855 |  |  |
|  |  |  |  |  | 30458601 | cg11594821 |  |  |
|  |  |  |  |  | 30458730 | cg21758773 |  |  |
| KHDRBS2 | KH Domain-Containing, RNA-Binding, Signal Transduction-Associated Protein 2 | It is a mammalian RNA-binding protein that regulates alternative splicing and influences both mRNA splice site selection and exon inclusion. It is methylated by protein arginine N-methyltransferase-1 in vivo. | SLM-1, Sam68-Like Mammalian Protein 1, SLM1, HSLM-1 | 6 | 62995876 | cg00472801 | 1stExon;5'UTR | chr6:62995855-62996228 |
|  |  |  |  |  | 62995963 | cg18239753 |  |  |
|  |  |  |  |  | 62996022 | cg16587616 |  |  |
|  |  |  |  |  | 62996119 | cg26715952 | TSS200 |  |
|  |  |  |  |  | 62996130 | cg22014661 |  |  |
| MARCH11 | Membrane associated ring-CH-type finger 11 | Modulates lysosomal degradation and delivery. | E3 Ubiquitin-Protein Ligase MARCH11, MARCH-XI, Membrane Associated Ring Finger 11, RNF226. | 5 | 16180048 | cg00339556 | TSS200 | chr5:16179064-16180420 |
|  |  |  |  |  | 16180055 | cg01791874 |  |  |
|  |  |  |  |  | 16180062 | cg17030173 |  |  |
|  |  |  |  |  | 16180068 | cg17712694 |  |  |
|  |  |  |  |  | 16180072 | cg16150752 |  |  |
|  |  |  |  |  | 16180076 | cg21901718 |  |  |
|  |  |  |  |  | 16180259 | cg18325622 | TSS1500 |  |
|  |  |  |  |  | 16180266 | cg23065934 |  |  |
| MIR129-2 | microRNA 1292 | MIR129-2 is a tumor suppressive miRNA methylated in lymphoid malignancies. Diseases associated with MIR129-2 include Retinoblastoma and Univentricular Heart. Found also to be hypermethylated in colorectal cancer cell lines. | Hsa-Mir-129-2, Mir-129-2, MIRN129-2, MIR-129b. | 11 | 43602845 | cg15556502 | TSS200 | chr11:43602545-43603215 |
|  |  |  |  |  | 43602847 | cg14416371 |  |  |
|  |  |  |  |  | 43602857 | cg14944647 |  |  |
|  |  |  |  |  | 43602879 | cg01939477 |  |  |
|  |  |  |  |  | 43602914 | cg16407471 |  |  |
|  |  |  |  |  | 43602920 | cg05376374 |  |  |
|  |  |  |  |  | 43602965 | cg03365311 | Body |  |
| NID2 | Nidogen 2 | Cell-adhesion protein involved in maintaining basement membrane structure. NID2 is methylated in human gastrointestinal cancer and also serves as an early detection methylated biomarker for oral SCC and bladder cancer. | Osteonidogen, Nidogen-2, NID-2. | 14 | 52535028 | cg25277187 | Body | chr14:52534581-52536722 |
|  |  |  |  |  | 52535178 | cg14897833 |  |  |
|  |  |  |  |  | 52535425 | cg07975778 |  |  |
|  |  |  |  |  | 52535758 | cg13592399 | 5'UTR;1stExon |  |
| NT5C3 | Cytosolic 5'-nucleotidase 3A | Encodes for a transferase which functions in nucleotide metabolic process. Nucleotidase which shows specific activity towards cytidine monophosphate (CMP). Mutations of this gene is associated with hemolytic anemia. | NT5C3A NT5C3, P5N1, UMPH1, HSPC233, 7-Methylguanosine Phosphate-Specific 5'-Nucleotidase, Cytosolic III, Pyrimidine 5'-Nucleotidase, P5'N-1, CN-III, Lupin, P5N-1, PN-I, P36, HUMP1, POMP, PSN1, UMPH. | 7 | 33080496 | cg05427639 | 1stExon; 5'UTR;  Body |  |
|  |  |  |  |  | 33080500 | cg25064331 |  |  |
|  |  |  |  |  | 33080571 | cg26306289 | TSS200  Body |  |
|  |  |  |  |  | 33080615 | cg06206957 |  |  |
| PAX1 | Paired box 1 | Frequent methylation in HNSCC. Shown to segregate normal and neoplastic cervical cancer. Gene is silenced by methylation in ovarian and cervical cancers and may be a tumor suppressor gene | Paired Box Gene 1, HUP48, Paired Domain Gene HuP48, HuP48, OFC2. | 20 | 21686273 | cg19054524 | TSS200 | chr20:21686199-21687689 |
|  |  |  |  |  | 21686282 | cg08448701 |  |  |
|  |  |  |  |  | 21686293 | cg01783070 |  |  |
|  |  |  |  |  | 21686308 | cg19079845 | 1stExon  5'UTR |  |
| PDE4B | Phosphodiesterase 4B | Hydrolyzes cAMP: a key regulator of many important physiological processes. May be involved in mediating central nervous system effects of therapeutic agents, such as; antidepressants, anti-asthmatic and anti-inflammatory agents. Associated diseases include Schizophrenia, bipolar disorder and ocular hypotension. | CAMP-Specific 3',5'-Cyclic Phosphodiesterase 4B, Phosphodiesterase 4B, CAMP-Specific (Phosphodiesterase E4 Dunce Homolog, Drosophila), DPDE4, Phosphodiesterase 4B, CAMP-Specific (Dunce (Drosophila)-Homolog Phosphodiesterase E4), Phosphodiesterase E4 Dunce Homolog (Drosophila), Phosphodiesterase 4B, CAMP-Specific, Dunce-Like Phosphodiesterase E4, PDEIVB, PDE32. | 1 | 66258687 | cg19754554 | 5'UTR  TSS200 | chr1:66258440-66258918 |
|  |  |  |  |  | 66258760 | cg00046625 |  |  |
|  |  |  |  |  | 66259081 | cg26963271 | 5'UTR |  |
|  |  |  |  |  | 66259084 | cg24637364 |  |  |
| PEX5L | Peroxisomal biogenesis factor 5 like | Trafficking of peroxisomal matrix proteins | TRIP8b, PEX5R, PXR2, Pex5p-Related Protein, PEX5RP, Pex5Rp, PXR2B. | 3 | 179754529 | cg02009585 | TSS200 | chr3:179754520-179755245 |
|  |  |  |  |  | 179754533 | cg23346462 |  |  |
|  |  |  |  |  | 179754603 | cg02119363 |  |  |
|  |  |  |  |  | 179754613 | cg13473356 |  |  |
|  |  |  |  |  | 179754615 | cg04894619 |  |  |
| PRRT1 | Proline Rich Transmembrane Protein 1 | Specific function unknown. | Dispanin Subfamily D Member 1, Interferon Induced Transmembrane Protein Domain Containing 7, C6orf31, DSPD1, NG5, Chromosome 6 Open Reading Frame 31, IFITMD. | 6 | 32119616 | cg17218813 | 1stExon  5'UTR | chr6:32118101-32118544 |
|  |  |  |  |  | 32119639 | cg12387154 |  |  |
|  |  |  |  |  | 32119685 | cg13035743 |  |  |
|  |  |  |  |  | 32119691 | cg12602633 |  |  |
|  |  |  |  |  |  |  |  |  |
| PTGDR | Prostaglandin D2 Receptor | Associated with nasal polyposis and also with allergic asthma in Caucasians and blacks but not in some other ethnicity such as the Latinos. | PGD2 Receptor, Prostanoid DP Receptor, PGD Receptor, PTGDR1, ASRT1, AS1, DP1, DP. | 14 | 52734286 | cg24989962 | TSS200 | chr14:52734207-52735486 |
|  |  |  |  |  | 52734325 | cg17929687 |  |  |
|  |  |  |  |  | 52734397 | cg02191312 |  |  |
|  |  |  |  |  | 52734525 | cg05302386 | 1stExon  5'UTR |  |
|  |  |  |  |  | 52734529 | cg09516965 |  |  |
| RYR2 | Ryanodine receptor 2 | Mediates cellular calcium release | Islet-Type Ryanodine Receptor, HRYR-2, ARVC2, VTSIP, ARVD2, RyR2, RyR. | 1 | 237205174 | cg07790615 | TSS1500 | chr1:237205126-237206644 |
|  |  |  |  |  | 237205295 | cg03422911 |  |  |
|  |  |  |  |  | 237205409 | cg18375860 |  |  |
|  |  |  |  |  | 237205950 | cg11657808 | Body |  |
|  |  |  |  |  | 237205999 | cg07914084 |  |  |
| SDCCAG8 | Serologically Defined Colon Cancer Antigen 8 | Protein coding gene involved in organizing the centrosome during interphase and mitosis. Associated diseases include: Bardet-Biedl Syndrome 16 and Senior-Loken Syndrome 7. | Centrosomal Colon Cancer Autoantigen Protein, Bardet-Biedl Syndrome 16, Antigen NY-CO-8, Nephrocystin 10, NPHP10, HCCCAP, CCCAP, Senior-Loken Syndrome 7, SLSN7, HSPC085, NY-CO-8, BBS16, CCCAP SLSN7. | 1 | 243646235 | cg16126286 | Body | chr1:243646394-243646888 |
|  |  |  |  |  | 243646318 | cg01550716 |  |  |
|  |  |  |  |  | 243646395 | cg27259271 |  |  |
|  |  |  |  |  | 243646402 | cg22699026 |  |  |
| SLCO4C1 | Solute Carrier Organic Anion Transporter Family Member 4C1 | It is a sodium-independent organic anion transporter known to be involved in spermatogenesis, cell differentiation and other transmembrane transport activity. It is associated with Eastern Equine Encephalitis. | Solute Carrier Family 21 Member 20; Organic Anion Transporter M1; SLC21A20; OATP-M1; OATP4C1; OATP-H; OATPX; Solute Carrier Organic Anion Transporter Family, Member 4C1; PRO2176. | 5 | 101632310 | cg19788741 | TSS200 | chr5:101632049-101632373 |
|  |  |  |  |  | 101632314 | cg22149516 |  |  |
|  |  |  |  |  | 101632321 | cg06480736 |  |  |
|  |  |  |  |  | 101632327 | cg11267955 |  |  |
|  |  |  |  |  | 101632341 | cg04621020 |  |  |
| SORCS3 | Sortilin related VPS10 domain containing receptor 3 | Frequently methylated in gastric cancer | VPS10 Domain Receptor Protein SORCS 3, KIAA1059, SORCS. | 10 | 106400824 | cg08495770 | TSS200 | chr10:106399567-106402812 |
|  |  |  |  |  | 106400880 | cg16787600 | 1stExon  5'UTR |  |
|  |  |  |  |  | 106401319 | cg10778841 | 1stExon |  |
|  |  |  |  |  | 106401479 | cg18326021 |  |  |
| ST6GALNAC5 | ST6 N-Acetylgalactosaminide Alpha-2,6-Sialyltransferase 5 | Involved in the biosynthesis of ganglioside GD1a from GM1b. Associated with disorders such as: Acute Hemorrhagic Conjunctivitis, Congenital Disorder of Glycosylation, Type Iif (CDG2F) and Miller Fisher Syndrome*.* | ST6 (Alpha-N-Acetyl-Neuraminyl-2,3-Beta-Galactosyl-1,3)-N-Acetylgalactosaminide Alpha-2,6-Sialyltransferase 5; Alpha-N-Acetylgalactosaminide Alpha-2,6-Sialyltransferase 5; ST6 GalNAc Alpha-2,6-Sialyltransferase 5; GalNAc Alpha-2,6-Sialyltransferase V, Sialyltransferase 7E, GD1 Alpha Synthase, ST6GalNAc V, ST6GalNAcV, SIAT7-E ,Alpha-N-Acetylneuraminyl 2,3-Betagalactosyl-1,3)-N-Acetyl Galactosaminide Alpha-2,6-Sialyltransferase E; ST6 Neuraminyl-2,3-Beta-Galactosyl-1,3)-N-Acetylgalactosaminide Alpha-2,6-Sialyltransferase 5, Alpha-N-Acetylgalactosaminide Alpha-2,6-Sialyltransferase V. | 1 | 77333138 | cg13823136 | TSS200 | chr1:77333111-77334534 |
|  |  |  |  |  | 77333159 | cg13463054 |  |  |
|  |  |  |  |  | 77333198 | cg06201642 | 1stExon;5'UTR |  |
|  |  |  |  |  | 77333229 | cg04077662 |  |  |
| TRIM31 | Tripartite Motif Containing 31 | TRIM31 is a RBCC protein with the ability to regulate cell proliferation negatively. It is overexpressed from the early stage of gastric adenocarcinoma. | RING-Type E3 Ubiquitin Transferase TRIM31, E3 Ubiquitin-Protein Ligase TRIM31, C6orf13, RNF, HCG1, HCGI. | 6 | 30079203 | cg21232488 | Body |  |
|  |  |  |  |  | 30079256 | cg27342919 |  |  |
|  |  |  |  |  | 30079265 | cg21809927 |  |  |
|  |  |  |  |  | 30079280 | cg08222513 |  |  |
| TRIO | Trio Rho Guanine Nucleotide Exchange Factor | Protein coding gene involved in re-organization on actin cytoskeleton, which is necessary for cell migration and growth. Associated diseases include Mental Retardation, Autosomal Dominant 44 and Loeys-Dietz Syndrome 2. | Triple Functional Domain (PTPRF Interacting), Triple Functional Domain Protein, PTPRF-Interacting Protein, ARHGEF23, MEBAS, MRD44, Tgat. | 5 | 14440492 | cg07866001 | Body |  |
|  |  |  |  |  | 14440991 | cg09115473 |  |  |
|  |  |  |  |  | 14441074 | cg22727783 |  |  |
|  |  |  |  |  | 14441261 | cg05860111 |  |  |
| VSTM2B | V-Set And Transmembrane Domain Containing 2B | Plays a role in the regulation of the early stage of white and brown pre-adipocyte cell differentiation. | V-Set And Transmembrane Domain-Containing Protein 2B. | 19 | 30016136 | cg18802754 | TSS1500 | chr19:30015781-30021367 |
|  |  |  |  |  | 30016147 | cg01464835 |  |  |
|  |  |  |  |  | 30016478 | cg02012703 |  |  |
|  |  |  |  |  | 30016511 | cg05395302 |  |  |
|  |  |  |  |  | 30017511 | cg04711162 | 1stExon |  |
| WDR17 | WD repeat domain 17 | Frequently altered in T cell malignancies | WD Repeat-Containing Protein 17, Epididymis Secretory Sperm Binding Protein. | 4 | 176987009 | cg11923920 | 1stExon  5'UTR | chr4:176986921-176987360 |
|  |  |  |  |  | 176987020 | cg08095852 |  |  |
|  |  |  |  |  | 176987174 | cg27486637 |  |  |
|  |  |  |  |  | 176987313 | cg08684639 | 5'UTR |  |
| ZIK1 | Zinc finger protein interacting with K protein 1 | Methylated biomarker for esophageal SCC. | Zinc Finger Protein Interacting With Ribonucleoprotein K, Zinc Finger Protein 762, ZNF762. | 19 | 58095518 | cg00800512 | TSS200 | chr19:58094739-58095764 |
|  |  |  |  |  | 58095581 | cg12060744 |  |  |
|  |  |  |  |  | 58095588 | cg01046104 |  |  |
|  |  |  |  |  | 58095595 | cg18579862 |  |  |
|  |  |  |  |  | 58095659 | cg26246807 | 5'UTR  1stExon |  |
| ZNF135 | Zinc finger protein 135 | DNA binding protein which functions in regulation of cell morphogenesis and organization of cytoskeleton. Associated with Causalgia disease. |  | 19 | 58570419 | cg08701621 | TSS200 | chr19:58570393-58571779 |
|  |  |  |  |  | 58570427 | cg06454760 |  |  |
|  |  |  |  |  | 58570454 | cg02473540 |  |  |
|  |  |  |  |  | 58570466 | cg09907936 |  |  |
| ZNF154 | Zinc finger protein 154 | Hypermethylation in 15 of 16 distinct cancer types from TCGA | Zinc Finger Protein 154 (PHZ-92), KIAA2003, PHZ-92. | 19 | 58220080 | cg03142586 | Body | chr19:58220189-58220517 |
|  |  |  |  |  | 58220295 | cg11294513 |  |  |
|  |  |  |  |  | 58220370 | cg05661282 | 5'UTR;1stExon |  |
|  |  |  |  |  | 58220494 | cg21790626 |  |  |
|  |  |  |  |  | 58220516 | cg27049766 |  |  |
|  |  |  |  |  |  |  |  |  |
| ZNF177 | Zinc finger protein 177 | Methylated in gastric and hepatocellular cancer | PIGX | 19 | 9473674 | cg13703871 | TSS200 | chr19:9473589-9474001 |
|  |  |  |  |  | 9473684 | cg08065231 |  |  |
|  |  |  |  |  | 9473688 | cg09578475 |  |  |
|  |  |  |  |  | 9473691 | cg07788092 |  |  |
| ZNF418 | Zinc Finger Protein 418 | Functions in DNA-binding transcription factor activity. Hypermethylated in Esophageal squamous cell carcinoma. | KIAA1956 | 19 | 58446669 | cg11998703 | 1stExon  5'UTR | chr19:58446336-58446800 |
|  |  |  |  |  | 58446745 | cg13668618 | TSS200 |  |
|  |  |  |  |  | 58446758 | cg12961842 |  |  |
|  |  |  |  |  | 58446770 | cg11788523 |  |  |
|  |  |  |  |  | 58446783 | cg15060012 |  |  |
|  |  |  |  |  | 58446787 | cg21444693 |  |  |
|  |  |  |  |  | 58446898 | cg18673377 |  |  |
| ZNF529 | Zinc finger protein 529 | No published information on this gene | KIAA1615 | 19 | 37096148 | cg25397945 | Body; 5'UTR  1stExon  TSS200 | chr19:37095680-37096589 |
|  |  |  |  |  | 37096321 | cg02587316 | TSS200  5'UTR |  |
|  |  |  |  |  | 37096323 | cg18630667 |  |  |
|  |  |  |  |  | 37096329 | cg05020604 |  |  |
| ZNF665 | Zinc Finger Protein 655 | Zinc finger proteins are generally involved in DNA binding and protein-protein interactions. ZNF655 may be involved in transcriptional regulation. | VIK, Vav-1 Interacting Kruppel-Like Protein, Vav-Interacting Krueppel-Like Protein, VIK-1 | 19 | 53696569 | cg11265561 | 1stExon  5'UTR | chr19:53696029-53696650 |
|  |  |  |  |  | 53696623 | cg12360029 | TSS200 |  |
|  |  |  |  |  | 53696642 | cg07686635 |  |  |
|  |  |  |  |  | 53696649 | cg01620580 |  |  |
| ZNF833 | Zinc Finger Protein 833, pseudogene | Non-annotated gene suspected to play a role in nucleic acid binding. | Putative zinc finger protein 833, ZNF833P, CTC-499B15.5 | 19 | 11784672 | cg26772540 | TSS200 | chr19:11785042-11785282 |
|  |  |  |  |  | 11784708 | cg25394203 |  |  |
|  |  |  |  |  | 11784731 | cg26590664 |  |  |
|  |  |  |  |  | 11784761 | cg21771200 |  |  |
| ZSCAN1 | Zinc Finger And SCAN Domain Containing 1 | Associated with Desbuquois dysplasia: short stature, joint laxity, developmental delay, hand anomalies, fusion protein with EV4 in prostate cancer. Hypermethylated in Cervical Cancer | SCAN1; CANT1 | 19 | 58545001 | cg14886059 | TSS1500 | chr19:58545115-58545897 |
|  |  |  |  |  | 58545122 | cg11312896 |  |  |
|  |  |  |  |  | 58545149 | cg21331821 |  |  |
|  |  |  |  |  | 58545160 | cg24368848 |  |  |
|  |  |  |  |  | 58545182 | cg25537993 |  |  |
|  |  |  |  |  | 58545285 | cg02344833 | TSS200 |  |
